# Swarming genetic algorithm: A nested fully coupled hybrid of genetic algorithm and particle swarm optimization

**DOI:** 10.1371/journal.pone.0275094

**Published:** 2022-09-23

**Authors:** Panagiotis Aivaliotis-Apostolopoulos, Dimitrios Loukidis

**Affiliations:** Department of Civil and Environmental Engineering, University of Cyprus, Nicosia, Cyprus; Torrens University Australia, AUSTRALIA

## Abstract

Particle swarm optimization and genetic algorithms are two classes of popular heuristic algorithms that are frequently used for solving complex multi-dimensional mathematical optimization problems, each one with its one advantages and shortcomings. Particle swarm optimization is known to favor exploitation over exploration, and as a result it often converges rapidly to local optima other than the global optimum. The genetic algorithm has the ability to overcome local extrema throughout the optimization process, but it often suffers from slow convergence rates. This paper proposes a new hybrid algorithm that nests particle swarm optimization operations in the genetic algorithm, providing the general population with the exploitation prowess of the genetic algorithm and a sub-population with the high exploitation capabilities of particle swarm optimization. The effectiveness of the proposed algorithm is demonstrated through solutions of several continuous optimization problems, as well as discrete (traveling salesman) problems. It is found that the new hybrid algorithm provides a better balance between exploration and exploitation compared to both parent algorithms, as well as existing hybrid algorithms, achieving consistently accurate results with relatively small computational cost.

## 1. Introduction

In recent years, there has been an increasing need for swift and accurate solutions to complex problems in many fields, such as science, engineering, manufacturing, finance etc., which has led to the development and implementation of a variety of modern mathematical optimization algorithms. With the constant rise in the computational power of CPUs, the use of heuristic algorithms is becoming increasingly more popular in recent decades. Heuristic optimization algorithms are particularly suitable for complex and computationally heavy multivariate problems that exhibit a plethora of local extrema. This is because, unlike gradient algorithms, they rely on the use of a random population of candidate solutions (“individuals” or “particles”) inside the search space. The goal of finding the best solution (global optimum) is pursued through an iterative procedure that considers a variety of interactions within the population. An effective heuristic algorithm is expected to ensure the quick and repeatable convergence to a satisfactory solution. As such, the creation of an efficient heuristic algorithm requires the balance of two major factors: exploration and exploitation. Exploitation is the algorithm’s ability to converge fast to a solution, whereas exploration is the ability to overcome local extrema successfully, thus reaching the global optimum.

Various heuristic algorithms have been proposed so far in the scientific literature, such as the Genetic Algorithm [1], Simulated Annealing [2], Tabu search [3], Particle Swarm Optimization [4], Ant Colony Optimization [5], Differential Evolution [6], Shuffled Frog-Leaping Algorithm [7], Invasive Weed Optimization [8], Artificial Bee Colony [9], Gravitational Search Algorithm [10], Grey Wolf Optimizer [11, 12], MTDE [13], NMPA [14]. Researchers have also combined different algorithms to produce hybrid versions possessing improved performance, e.g. ANGEL [15], PSOGSA [16], SFLA-IWO [17], GGWO [18], WOA [19], AOA [20], m-SCBOA [21], FRCSA [22]. The present study focuses on the Genetic Algorithm and the Particle Swarm Optimization, and their combination into hybrid algorithms.

Evolutionary algorithms are a sub-set of heuristic algorithms, with the Genetic Algorithm (GA) introduced by Holland [1] being one of the most used and well known. GA is based on the principles of natural evolution and the concept of the survival of the fittest. The sets of optimization variables are treated as chromosomes that are subjected to the three main genetic operators, namely selection, crossover and mutation, in order to achieve a better solution with each subsequent generation. GA favors exploration over exploitation and, as a consequence, its convergence is often slow [23].

Particle Swarm Optimization (PSO) is a social evolutionary algorithm developed by Kennedy & Eberhart [4] based on swarm intelligence. It was inspired by the social behavior of flock of birds and school of fishes. The sets of optimization variables are treated as particles in the search space, where the position of a particle is a solution to the studied problem. Each particle is moving in the search space iteration by iteration according to its score, i.e. the corresponding value of the objective function (personal aspect), relative position to other particles (social aspect) and inertia (change of location in previous iteration). PSO is a fast-converging algorithm favoring exploitation over exploration, but as a result the algorithm is susceptible to getting trapped in local extrema, thus failing to yield the true (global) optimal solution.

In order to alleviate the inherent shortcomings of each of the individual methods, researchers have proposed several hybrid algorithms by combining GA and PSO operations, with the basic idea being the achievement of better balance between exploration and exploitation [16, 23–28]. In these algorithms, GA is usually responsible for the exploration, while PSO focuses in providing improved convergence speed. The hybrid algorithms differ between each other in the details of the coupling of the GA and PSO, e.g. whether a method is nested inside the other (and which) or they are conducted in parallel, and how they interact (i.e. how the outcome of one method influences the computations of the other). For example [25], proposed a hybrid form of PSO with the incorporation of GA’s mutation property, thus providing PSO with an escape route from local extrema. A different adaptation was proposed in [28] with coupled parallel GA and PSO operations. Jeong et al. [23] applied a similar parallel combination to multi-objective optimization. Herein, a new hybrid approach is proposed combining GA and PSO. Its novelty lies on that the PSO is nested inside the GA and applied to only a part of the GA population, aiming to achieve a better balance between exploration and exploitation compared to existing hybrid methods. The performance of the new approach is tested and compared with that of parent algorithms and two existing hybrid algorithms for two sets of continuous benchmark problems, as well as a set of discrete (traveling salesman) problems.

## 2. Algorithms

In this section, we first provide the framework for GA and PSO in the form they are employed in this study. Subsequently, we present the proposed hybrid algorithm, along with two existing hybrid algorithms from which inspiration was drawn [25, 26].

### 2.1 Genetic algorithm

Being among the earliest heuristic methods, genetic algorithms have seen widespread application in many scientific fields. In GA, each candidate solution **x**_*j*_ = {*x*_1,*j*_,*x*_2,*j*_,…,*x*_*D*,*j*_} to the problem is termed an “individual” or “chromosome”, with the free (optimization) scalar variables *x*_*i*_ being considered “genes”. The size *M* of the population of individuals to be the considered in the search space at the start of the solution process is set by the user. The algorithm begins by randomly creating sets of genes for the *M* individuals and then evaluating the objective function *f*(**x**_*j*_) for each individual. One of the individuals (the global best) would yield the best current solution *f*(**x**_GB_), i.e. min{*f*(**x**_1_),…,*f*(**x**_M_)} for minimization problems or max{*f*(**x**_1_),…,*f*(**x**_M_)} for maximization problems.

Since the basic concept of the algorithm is the “survival of the fittest”, a fitness level *L*_*FT*_ is assigned to each individual *j*, representing the suitability of the solution compared to its peers:

$$L_{FT,j}=\frac{f(x_{GB})}{f(x_{j})}$$ (1)

Before the next iteration of the algorithm, a group of individuals (either randomly selected or targeted) is subjected to random mutations of the chromosomes and crossover breeding.

The mutations are instrumental for avoiding convergence to a sub-optimal solution by keeping the individuals uncluttered. For continuous problems, mutations can be done by adding to (or subtracting from) *x*_i_ a random percentage of the width *W*_i_ of the search space in the corresponding (i^th^) dimension as follows:

$$x_{i,j}^{\left(iter+1\right)}=x_{i,j}^{\left(iter\right)}+rand_{\mathrm{mu}}\cdot R\cdot W_{i}$$ (2)

where *rand*_mu_ are random real numbers between -1 and 1, and *R* is the maximum possible percentage change, which is usually a parameter selected by the user. Eq (2) applies provided that the mutated individual remains inside the search space and any optimization constraints are satisfied. In case $x_{i,j}^{\left(iter+1\right)}$ computed using Eq (2) falls outside the search space, its value is set equal to the closest limiting value (i.e. max *x*_*i*_ or min *x*_*i*_).

Crossover provides the means for convergence by combining two individuals to create a new one (offspring) to be used in the next iteration of the algorithm. For continuous problems, an offspring may be created through arithmetic crossover [29] as a linear interpolation (it can be seen also as a weighted average) between two existing solutions (parents), as follows:

$$x_{\left(offspring\right)}=rand_{cr}\circ x_{\left(parent1\right)}+(1-rand_{cr})\circ x_{\left(parent2\right)}$$ (3)

where **rand**_cr_ is a vector of random numbers between 0 and 1. In order to completely replace the two parents before the next iteration, a second offspring is created by swapping the weight factors **rand**_cr_ and (1-**rand**_cr_).

**Fig 1:**
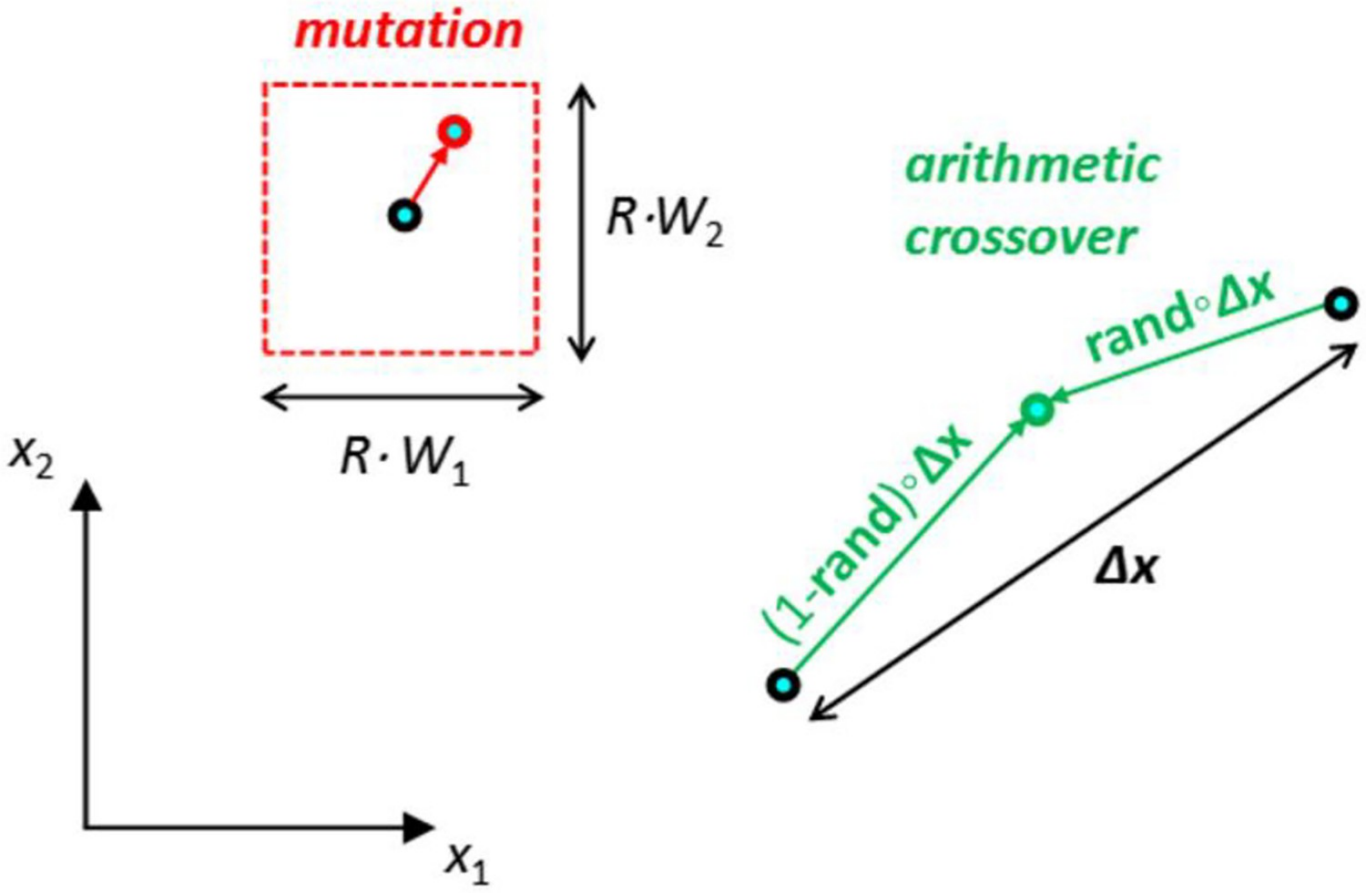
Schematic representation of GA mutation and crossover.

Elitist selection is often employed in GA, i.e. a proportion of best individuals of the population (elite group) is retained to the next generation. More recently, in order to enhance the exploitation capabilities of GA, elitist crossover is also considered, i.e. crossover is focused in a group of best performing individuals [30, 31]. Fig 1 shows schematically the aforementioned mutation and crossover processes.

**Fig 2:**
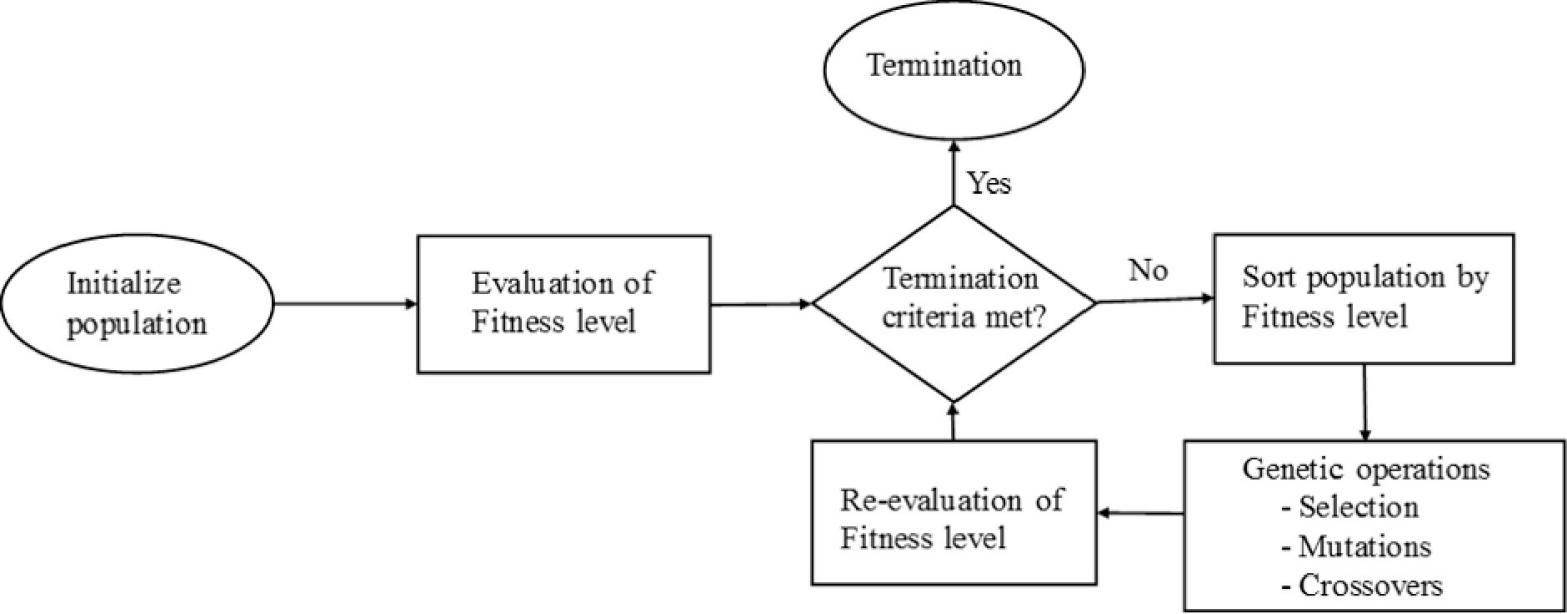
Typical flow chart for genetic algorithm.

The above procedure is repeated iteratively until a set of specified stopping criteria are met (e.g. exceedance of a maximum number of iterations or the global best remains constant for a given number of iterations). The sequence of these steps is shown in the form of flow chart in Fig 2.

### 2.2 Particle swarm optimization

PSO operates by moving the individuals (particles) in the multi-dimensional search space in a geometric manner. The method starts by generating a preset number (*M*) of particles at random positions. Each particle *j* has a vector indicating its current position (**x**_*j*_) and a vector symbolizing its spatial velocity **v**_*j*_, i.e. the change of particle position between successive iterations. Every particle has also a “memory” of its best position (**x**_PB,*j*_), which is kept along with the global best position (**x**_GB_) of the entire population. Both **x**_PB,*j*_ and **x**_GB_ are acting as attractors for particle *j*. At the start of the algorithm, the particles’ initial velocities are set equal to zero. Each particle velocity at subsequent steps is set to be directly proportional to 1) its previous velocity (inertial component), 2) the distance of the current particle position from the global best (social component) and 3) the distance from its personal best (personal component), according to the following equation:

$$v_{j}{}^{\left(iter+1\right)}=w\cdot v_{j}{}^{\left(iter\right)}+c_{1}\cdot rand_{1}\circ (x_{GB}^{\left(iter\right)}-x_{j}{}^{\left(iter\right)})+c_{2}\cdot rand_{2}\circ (x_{PB,j}^{\left(iter\right)}-x_{j}{}^{\left(iter\right)})$$ (4)

where **rand**_1_ and **rand**_2_ are vectors of random numbers between 0 and 1, and *c*_1_ and *c*_2_ are constants scaling the “social” and “personal” gravitational components, respectively. The factor *w* is an “inertia” coefficient controlling the influence of the current velocity value to that of the next iteration. Once the new velocity is stablished using Eq (4), the new particle position is calculated as

$$x_{j}{}^{\left(iter+1\right)}=x_{j}{}^{\left(iter\right)}+v_{j}{}^{\left(iter+1\right)}$$ (5)

**Fig 3:**
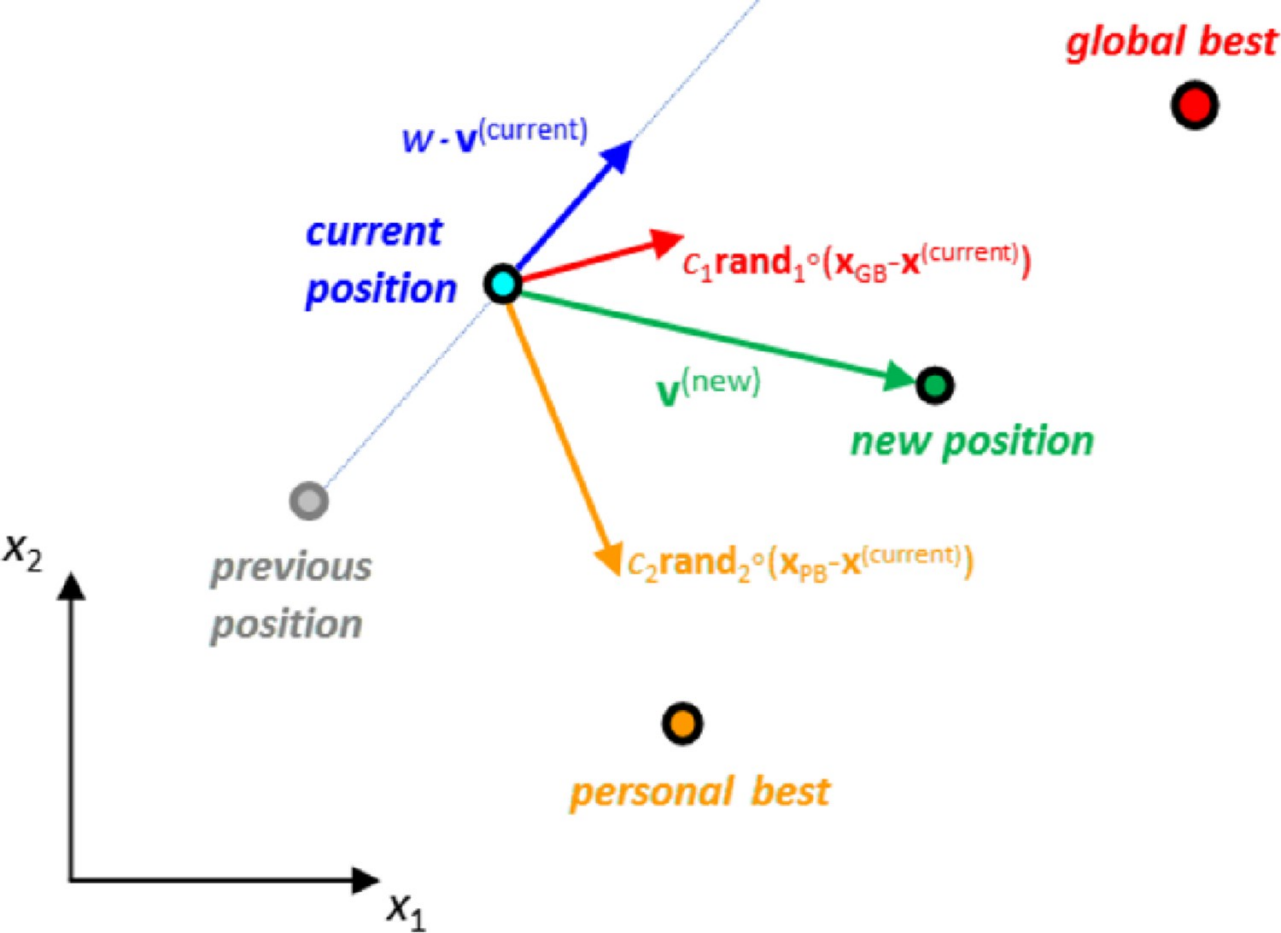
Schematic representation of PSO velocity components.

Fig 3 shows a schematic representation of the inertial, social and personal velocity components.

**Fig 4:**
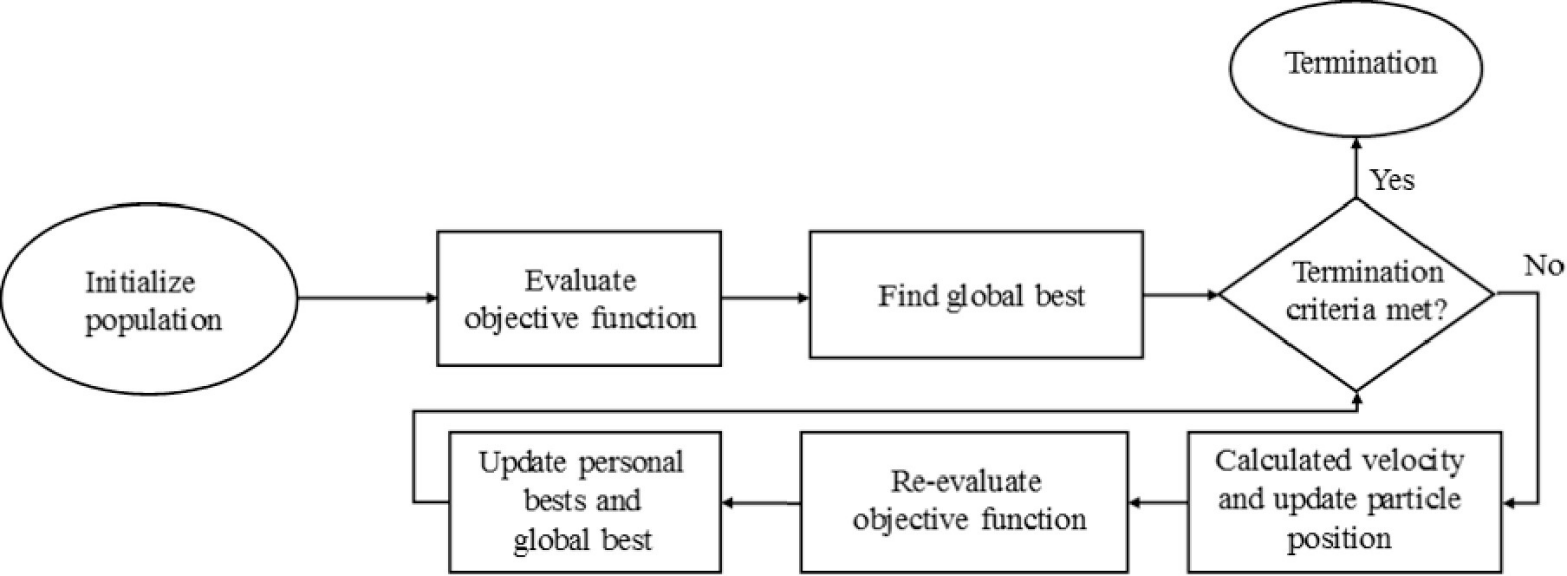
Flow chart for particle swarm optimization.

This procedure is repeated iteratively until a set of specified convergence and stopping criteria are met (Fig 4). Common practice is to set the inertial factor *w* to decrease (e.g. linearly or exponentially) as the iteration number increases, a technique often referred as “damping”, in order to facilitate convergence [32]. Herein, we set *w* to decrease linearly with the number of iterations:

$$w=w_{\max}-(w_{\max}-w_{\min})\frac{iter}{maxiter}$$ (6)

where *w*_max_ and *w*_min_ are the maximum (initial) and minimum values of the inertial factor, and *maxiter* is the maximum allowed number of iterations. For the PSO to be able to achieve convergence, the *w*_min_ needs to be well below 1.0.

### 2.3 Hybrid algorithms

#### 2.3.1 HPSOM

The standard PSO is formulated in such a way that convergence to a solution is guaranteed, provided that *w* is set initially or becomes eventually less than unity. This is because the particles tend to converge with successive iterations to a global best, where both social and personal velocity components become zero. However, there is no guarantee that this solution is the global optimum and not a local one. To alleviate this major shortcoming and in order to boost the exploration prowess of the PSO algorithm, Esmin et al. [26] proposed the incorporation of particle mutations. In their algorithm, which is called Hybrid Particle Swarm Optimizer with Mutation (HPSOM), in each iteration once PSO calculations are completed, a group of particles is randomly chosen to undergo mutation (Eq 2). HPSOM can be seen as connecting the PSO and GA processes in series, with PSO preceding GA and with absence of crossovers.

#### 2.3.2 PGPHEA

**Fig 5:**
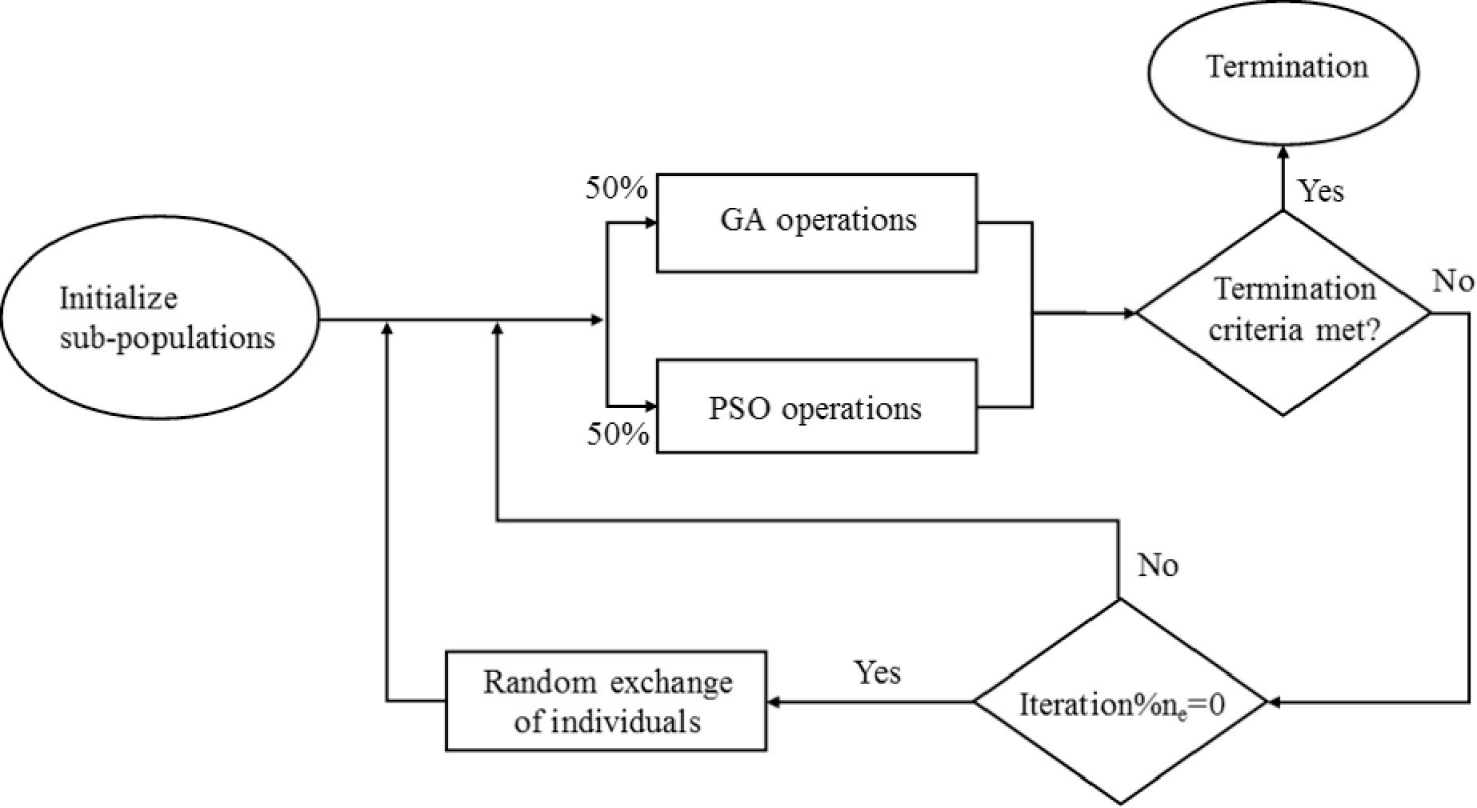
Flow chart for PGPHEA algorithm.

Shi et al. [28] proposed an algorithm combining PSO and GA in parallel, named PSO-GA parallel hybrid evolutionary algorithm (PGPHEA). The algorithm starts by setting an initial population divided in two random sub-populations, one undergoing GA computations while the other PSO. For a user-defined number of iterations (*n*_e_), each sub-population is undergoing its optimization algorithm without any interaction. Every *n*_e_ iterations, there is an exchange of a fixed proportion of randomly selected individuals between the two sub-populations. After each exchange, the global bests are redefined in each group and the process is repeated until reaching the set termination criteria (Fig 5). Herein, the two sub-populations are set to be of equal size, following [23]. Moreover, once the exchanged individuals enter PSO, their personal best is set equal to themselves and the inertial velocity component is set equal to zero.

## 3. Swarming genetic algorithm

**Fig 6:**
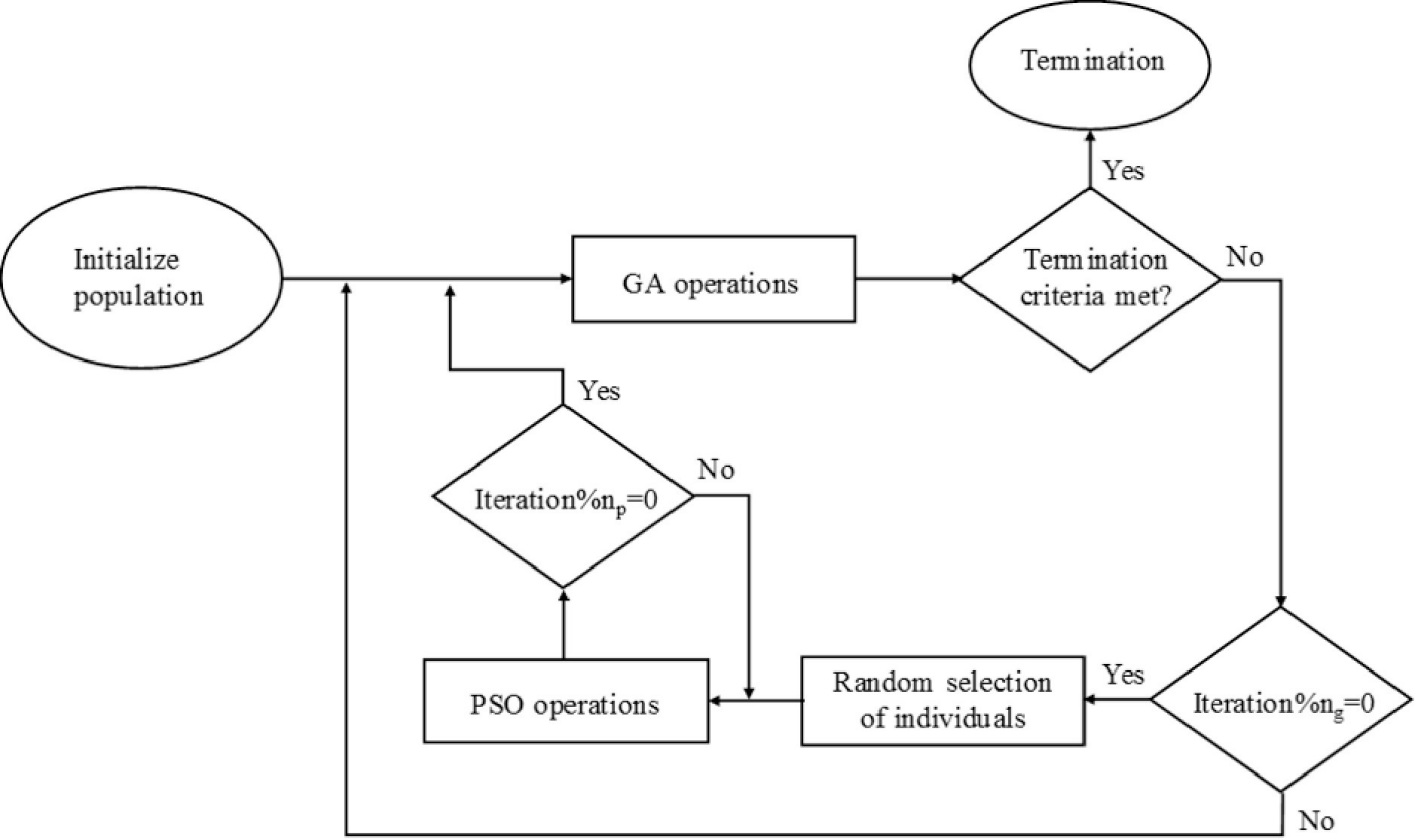
Flow chart for the proposed hybrid algorithm.

**Fig 7:**
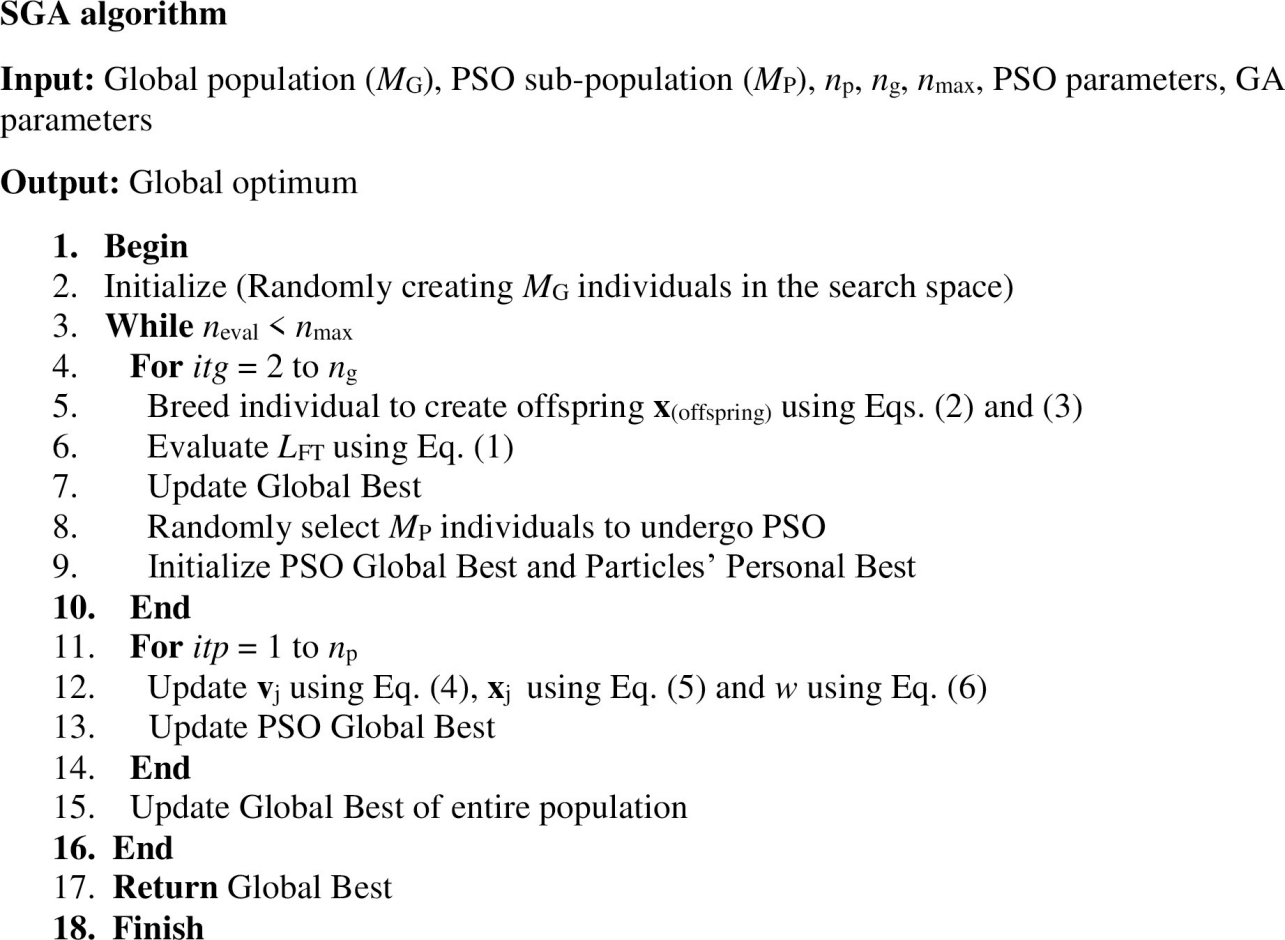
Pseudocode of the proposed hybrid algorithm.

The main idea of the proposed hybrid algorithm is to incorporate the fast convergence capabilities of PSO in the strongly exploratory GA. The hybrid algorithm formulated herein, named Swarming Genetic Algorithm (SGA), nests PSO loops inside external loops of GA, as shown in Fig 6 and in the form of pseudo-code (Fig 7). Every *n*_g_ iterations of GA, a small sub-group (*M*_P_) of the global population (*M*_G_) is randomly selected to undergo PSO calculations for a specified number of iterations *n*_p_. Each time a block of *n*_p_ PSO iterations starts, the initial particle velocities are set equal to zero and the personal bests are reset to the current position of the corresponding particles, while the global best is re-evaluated based on the outcome of the preceding GA calculations for the current PSO sub-population. In other words, PSO does not retain a memory of the previous block of PSO calculations. After *n*_p_ iterations, the PSO sub-group is re-introduced to the overall population, which then undergoes new GA operations of mutations and crossovers. The sub-group that undergoes PSO usually converges rapidly to a solution, but given that the rest of the population is unaffected by the nested PSO, the algorithms maintain the strong exploratory character of the standard GA. Computations terminate once certain criteria are met, e.g. the cumulative number of evaluations of the objective function *n*_eval_ reaches a preset limit *n*_max_. From Figs 6 and 7, it can be seen that the added complexity of nesting PSO calculations in GA is minimal and, as it will be shown in the following section, the proposed hybrid algorithm is competitive regarding its computational cost.

## 4. Benchmark tests

In this section, the performance of SGA is compared against GA [1], PSO [4], HPSOM [26] and PGPHEA [28] for continuous as well as discrete (traveling salesman) single-objective optimization problems, both in terms of effectiveness in finding the global optimum and computational time efficiency.

### 4.1 Continuous problems

Two sets of continuous single-objective optimizations problems are considered herein. The first (Set A) contains 14 standard optimization functions with dimensions *D* (i.e. number of optimization variables) in the 2 to 30 range. The second set (Set B) contains the single-objective optimization problems of the CEC 2017 benchmark suite [33, 34] with *D* set equal to 50. All the problems of the second set are constrained and more demanding than those of the first set. Due to the probabilistic nature of the heuristic algorithms, each time a problem is solved with a given algorithm a different result may be produced. Hence, each algorithm was tested 100 consecutive times for each optimization problem. The performance of the algorithms is compared based on various metrics, such as the average and maximum errors, the Overall Effectiveness (OE) [35], and then ranked accordingly. The results are also analyzed using two statistical tests i.e., the Wilcoxon signed-rank test [16, 35], with the hypothesis that SGA provides smallest error, and the Friedman test.

The overall population size for all methods was equal to 100. The algorithmic parameters for PSO are set *c*_1_ = *c*_2_ = 2, *w*_max_ = 1 and *w*_min_ = 0.001. Moreover, a maximum limit was set to the particle velocity equal to 50% of the width of the search space. For the hybrid algorithms, the algorithmic parameters for the PSO component are the same as above, except in PGPHEA where they were increased to *w*_max_ = 2 and *w*_min_ = 0.01, as trials showed that this improves the performance of PGPHEA (S1 Table in S1 File). For the standard (non-hybridized) GA, an elite group of 30% of the population is isolated in order to undergo elite crossover, while 10% of the population undergoes mutation and 60% undergoes crossover, both randomly selected. In the GA component of the hybrid algorithms, 20% of the population undergoes mutations, 60% is derived from crossovers, and 20% is isolated for elite crossover operations. For PGPHEA, the number of iterations for a population exchange to happen is *n*_e_ = 100 steps. In SGA, in every iteration (*n*_g_ = 1) of GA (external loop) 20% of the population is randomly selected to undergo *n*_p_ = 100 PSO iterations (internal loop). For HPSOM, 20% of the population is set to undergo mutation, as it was found that higher percentages caused the algorithm to become unstable. The parameter *maxiter* in Eq (6) of PSO operations was set equal to *n*_p_ and *n*_e_ for SGA and PGPHEA, respectively. For PSO and HPSOM, *maxiter* = 2000.

The above algorithmic parameters and settings are considered as optimal and were established via several preliminary trial runs for the whole sets of continuous test functions that are considered herein. All calculations were performed on an Intel^®^ i7-11800H processor using scripts programmed in Matlab.

#### 4.1.1 Set A

The continuous problems of Set A are standard benchmarking optimization functions (*f*_1_-*f*_14_) [28, 36, 37] and are shown in Table 1, along with their search spaces and the number *D* of optimization variables (dimensions).

**Table 1:** Continuous benchmark problems of Set A.

f	Search Space	*D*	Optimization Problem	Function Name
f_1_	[–100,100]	30	$\min f(x)=\sum_{i=1}^{N-1}\left[{\left(x_{i+1}-x_{i}^{2}\right)}^{2}+{\left(x_{i}-1\right)}^{2}\right]$	Rosenbrock
f_2_	[–600,600]	30	$\min f(x)=\sum_{i=1}^{N}\frac{x_{i}^{2}}{4000}-\prod_{i=1}^{d}\cos(\frac{x_{i}}{\sqrt{i}})+1$	Griewank
f_3_	[–10,10]	30	$\min f(x)=10N+\sum_{i=1}^{N}\left[x_{i}^{2}-10\cos(2\pi x_{i})\right]$	Rastrigin
f_4_	[-32.768,32.768]	30	$\begin{array}{l} \min f(x)=-20\exp(-0.2\sqrt{\frac{1}{N}\sum_{i=1}^{N}x_{i}^{2}})\\ -\exp(\frac{1}{N}\sum_{i=1}^{N}\cos(2\pi x_{i}))+20+\exp(1) \end{array}$	Ackley
f_5_	[-65.536,65.536]	2	$\begin{array}{l} \min f(x)={\left(0.002+\sum_{i=1}^{25}\frac{1}{i+{\left(x_{1}-a_{1i}\right)}^{6}+{\left(x_{2}-a_{2i}\right)}^{6}}\right)}^{-1}\\ a=\left(\begin{array}{cccccccccc} -32 & -16 & 0 & 16 & 32 & -32 & \ldots & 0 & 16 & 32\\ -32 & -32 & -32 & -32 & -32 & -16 & \ldots & 32 & 32 & 32 \end{array}\right) \end{array}$	De Jong
f_6_	[0,1]	6	$\begin{array}{l} \min f(x)=-\sum_{i=1}^{4}c_{i}\exp(-\sum_{j=1}^{6}a_{ij}{\left(x_{j}-p_{ij}\right)}^{2})\\ a=\left(\begin{array}{cccccc} 10 & 3 & 17 & 3.5 & 1.7 & 8\\ 0.05 & 10 & 17 & 0.1 & 8 & 14\\ 3 & 3.5 & 1.7 & 10 & 17 & 8\\ 17 & 8 & 0.05 & 10 & 0.1 & 14 \end{array}\right)\\ c=(\begin{array}{cccc} 1 & 1.2 & 3 & 3.2 \end{array})\\ p=\left(\begin{array}{cccccc} 0.1312 & 0.1696 & 0.5569 & 0.0124 & 0.8283 & 0.5886\\ 0.2329 & 0.4235 & 0.8307 & 0.3736 & 0.1004 & 0.9991\\ 0.2348 & 0.1415 & 0.3522 & 0.2883 & 0.3047 & 0.6650\\ 0.4047 & 0.8828 & 0.8732 & 0.5743 & 0.1091 & 0.0381 \end{array}\right) \end{array}$	Hartmann
f_7_	[–50,50]	3	$\begin{array}{l} \max f(x)=x_{1}^{2}+x_{2}^{2}+x_{3}^{2}\\ s.t.\;g_{A}(x)=4{\left(x_{1}-0.5\right)}^{2}+2{\left(x_{2}-0.2\right)}^{2}\\ \;+x_{3}^{2}+0.1x_{1}x_{2}+0.2x_{2}x_{3}-16\leq 0\\ \;g_{B}(x)=2-2x_{1}^{2}-x_{2}^{2}+2x_{3}^{2}\leq 0 \end{array}$	[25]
f_8_	[–50,50]	2	$\begin{array}{l} \max f(x)=-{\left(x_{1}-2\right)}^{2}-{\left(x_{2}-1\right)}^{2}\\ s.t.\;g_{A}(x)=x_{1}-2x_{2}+1=0\\ \;g_{B}(x)=(x_{1}^{2}/4)+x_{2}^{2}-1\leq 0 \end{array}$	[25]
f_9_	[–100,100]	30	$\min f(x)=\sum_{i=1}^{N}x_{i}^{2}$	Spherical
f_10_	[0,10]	2	$\begin{array}{l} \min f(x)=\sum_{i=1}^{5}c_{i}\exp(-\frac{1}{\pi }\sum_{j=1}^{N}{\left(x_{j}-A_{ij}\right)}^{2})\cos\left(\pi \sum_{j=1}^{N}{\left(x_{j}-A_{ij}\right)}^{2}\right)\\ A=\left(\begin{array}{cc} 3 & 5\\ 5 & 2\\ 2 & 1\\ 1 & 4\\ 7 & 9 \end{array}\right),c=(\begin{array}{ccccc} 1 & 2 & 5 & 2 & 3 \end{array}) \end{array}$	Langermann
f_11_	[–512,512]	2	$\begin{array}{l} \min f(x)=-(x_{2}+47)\sin\left(\sqrt{\left|x_{2}+\frac{x_{1}}{2}+47\right|}\right)\\ -x_{1}\sin(\sqrt{\left|x1-(x_{2}+47)\right|}) \end{array}$	Eggholder
f_12_	[–100,100]	2	$\min f(x)=-\cos(x_{1})\cos(x_{2})\exp(-{\left(x_{1}-\pi \right)}^{2}-{\left(x_{2}-\pi \right)}^{2})$	Easom
f_13_	[–10,10]	2	$\min f(x)=(\sum_{i=1}^{5}i\cos((i+1)x_{1}+i))(\sum_{i=1}^{5}i\cos((i+1)x_{2}+i))$	Shubert
f_14_	[–500,500]	20	$\min f(x)=418.9829N-\sum_{i=1}^{N}x_{i}\sin(\sqrt{x_{i}})$	Schwefel

The global optima for the above benchmark problems are shown in Table 2. Table 1 contains mostly unconstrained minimization problems, with the exception of *f*_7_ and *f*_8_ [28] which pertain to constrained maximization. In order to convert the latter to unconstrained minimization problems (to make the studied cases more homogenous) with global optimum equal to zero, the following conversion was implemented:

$$f_{7}^{*}(x)=100-\frac{1}{|11.68-(f_{7}(x)+pen_{7})|+0.01}$$ (7A)

$$f_{8}^{*}(x)=100-\frac{1}{|-1.3777-(f_{8}(x)+pen_{8})|+0.01}$$ (7B)

$$pen_{7}=1000{\langle g_{A,7}\rangle }^{2}+1000{\langle g_{B,7}\rangle }^{2}$$ (8A)

$$pen_{8}=1000{\left(g_{A,8}\right)}^{2}+1000{\langle g_{B,8}\rangle }^{2}$$ (8B)

where *pen*_7_ and *pen*_8_ are penalty terms introducing in the objective functions the enforcement of the respective constraints in the form used in [28], and < > are the Macauley brackets (i.e. <x> = x for x≥0, otherwise = 0). With this transformation, the search range for the optimization variables of the modified functions *f*_7_* and *f*_8_* is 0 to 100.

**Table 2:** Global optima for objective functions *f*_1_ –*f*_14_.

Function	Global optimum
*f*_1_	0
*f*_2_	0
*f*_3_	0
*f*_4_	-2.60E+89
*f*_5_	0.998004
*f*_6_	-3.32237
*f*_7_*	0
*f*_8_*	0
*f*_9_	0
*f*_10_	-4.15581
*f*_11_	-959.641
*f*_12_	-1
*f*_13_	-186.731
*f*_14_	0

**Fig 8:**
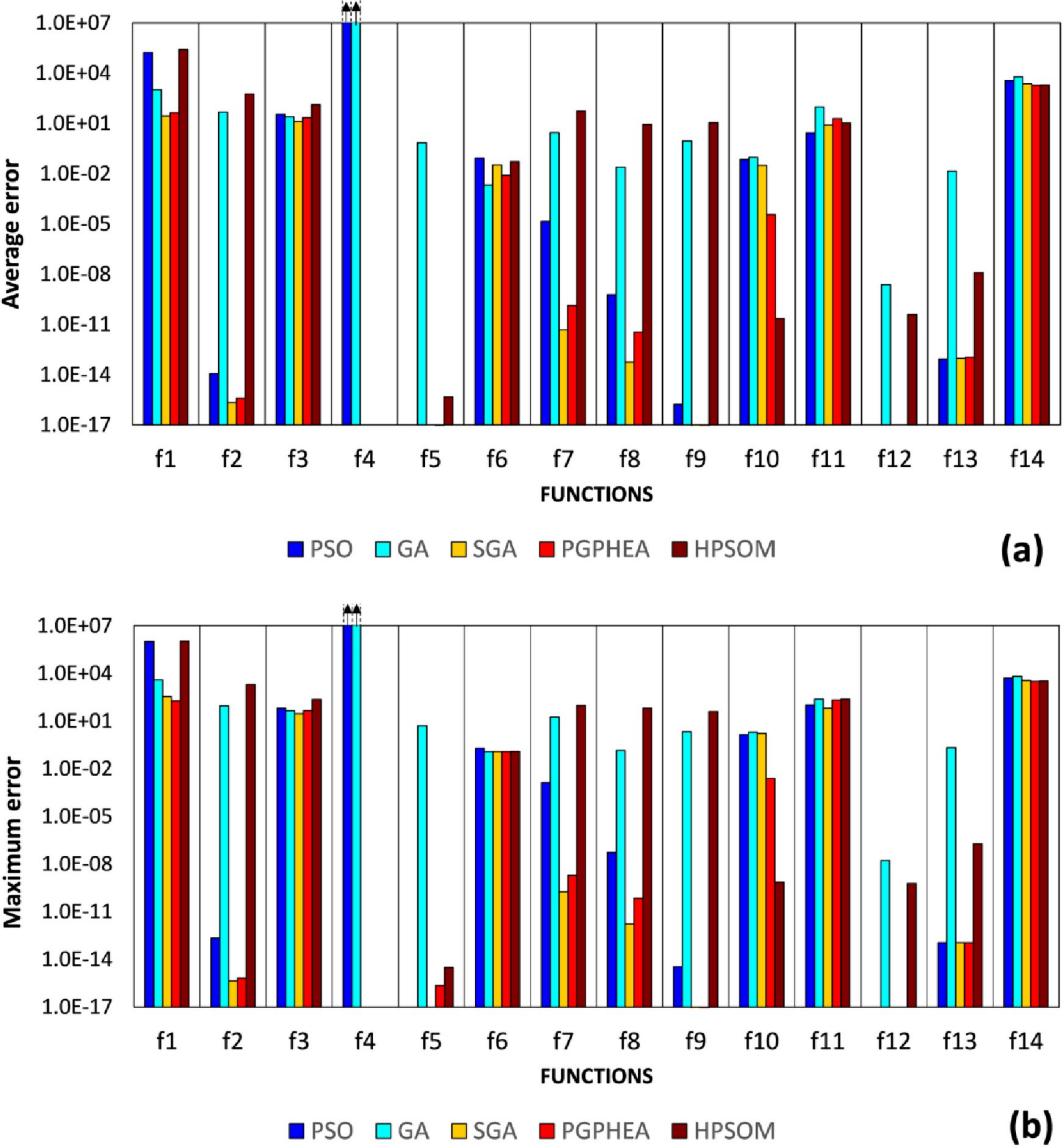
Comparison of algorithmic performance in solving the continuous problems of Set A: a) average error, b) maximum error in predicting the global optimum.

For all algorithms, computations are terminated once the cumulative number of evaluations of the objective function exceeds 40020 (termination criterion). The average error and maximum error from the 100 consecutive runs are shown in Tables 3 and 4, respectively, where grey shading marks the best performing algorithms in each case. These results are also compared graphically in Fig 8.

**Table 3:** Comparison of average error in finding the global optimum of functions *f*_1_-*f*_14_.

Function	PSO	GA	SGA	PGPHEA	HPSOM
*f*_1_	1.72E+05	1.03E+03	2.81E+01	4.30E+01	2.52E+05
*f*_2_	1.17E-14	4.68E+01	2.15E-16	3.86E-16	5.60E+02
*f*_3_	3.42E+01	2.47E+01	1.29E+01	2.22E+01	1.27E+02
*f*_4_	5.21E+88	2.60E+89	0.00E+00	0.00E+00	0.00E+00
*f*_5_	0.00E+00	7.11E-01	0.00E+00	8.88E-18	6.97E-16
*f*_6_	8.39E-02	2.06E-03	3.31E-02	7.98E-03	5.19E-02
*f*_7_	1.38E-05	2.80E+00	4.73E-12	1.32E-10	5.28E+01
*f*_8_	5.87E-10	2.41E-02	5.68E-14	3.50E-12	8.66E+00
*f*_9_	1.68E-16	8.85E-01	2.38E-48	5.89E-30	1.10E+01
*f*_10_	6.83E-02	9.27E-02	3.09E-02	3.54E-05	2.32E-11
*f*_11_	2.67E+00	9.56E+01	7.82E+00	1.92E+01	1.02E+01
*f*_12_	0.00E+00	2.32E-09	0.00E+00	0.00E+00	3.88E-11
*f*_13_	8.38E-14	1.42E-02	9.35E-14	1.10E-13	1.26E-08
*f*_14_	3.66E+03	6.01E+03	2.29E+03	1.90E+03	1.92E+03
***W/T/L***	**2/2/10**	**1/0/13**	**6/3/5**	**1/2/11**	**1/1/12**
***OE%***	**28.57%**	**7.14%**	**64.29%**	**21.43%**	**14.29%**

**Table 4:** Comparison of maximum error in finding global optimum of functions *f*_1_-*f*_14_.

Function	PSO	GA	SGA	PGPHEA	HPSOM
*f*_1_	1.00E+06	3.96E+03	3.49E+02	1.88E+02	1.08E+06
*f*_2_	2.18E-13	9.15E+01	4.44E-16	6.66E-16	1.99E+03
*f*_3_	6.37E+01	4.48E+01	2.89E+01	4.58E+01	2.26E+02
*f*_4_	2.60E+89	2.60E+89	0.00E+00	0.00E+00	0.00E+00
*f*_5_	0.00E+00	5.10E+00	0.00E+00	2.22E-16	3.11E-15
*f*_6_	1.92E-01	1.19E-01	1.19E-01	1.19E-01	1.23E-01
*f*_7_	1.36E-03	1.79E+01	1.80E-10	1.98E-09	9.50E+01
*f*_8_	5.42E-08	1.39E-01	1.66E-12	7.11E-11	6.44E+01
*f*_9_	3.49E-15	2.10E+00	1.18E-46	1.42E-28	3.96E+01
*f*_10_	1.41E+00	1.96E+00	1.66E+00	2.41E-03	7.34E-10
*f*_11_	1.01E+02	2.44E+02	6.51E+01	2.08E+02	2.41E+02
*f*_12_	0.00E+00	1.67E-08	0.00E+00	0.00E+00	5.88E-10
*f*_13_	1.14E-13	2.10E-01	1.14E-13	1.14E-13	1.82E-07
*f*_14_	5.14E+03	6.37E+03	3.57E+03	3.10E+03	3.34E+03
***W/T/L***	**0/3/11**	**0/0/14**	**6/5/3**	**2/4/8**	**1/1/12**
***OE%***	**21.43%**	**0.00%**	**78.57%**	**42.86%**	**14.29%**

It can be seen that SGA exhibits the best OE (64.29%), while there are significant statistical differences based on the Friedman test (*p*-value = 7.6828e-04). Nonetheless, it should be noted that the Wilcoxon test (Table 5) did not find significant differences between SGA and PGPHEA for the specified significance level. Moreover, PGPHEA yields the smaller errors than SGA in problem *f*_10_ and *f*_14_. In most of the cases (most notably *f*_1_ and *f*_4_), PSO gets trapped in local optima due to its limited exploration capabilities. Among PGPHEA and HPSOM, PGPHEA appears to achieve better convergence than HPSOM (Table 6). It is also interesting to note that in certain cases (most notably *f*_2_, *f*_7_, *f*_8_, *f*_9_) HPSOM yields larger errors than PSO. This indicates that the frequent mutation imposed on the PSO particles was making the algorithm less efficient for the specific functions.

**Table 5:** Wilcoxon signed-rank-test (*p* ≥ 0.05) for average error in continuous problems of Set A.

SGA vs.	PSO	GA	PGPHEA	HPSOM
p-value	1.05E-02	4.27E-04	1.90E-01	1.99E-02
Significant	Yes	Yes	No	Yes

**Table 6:** Algorithm Ranking for continuous problems of Set A.

Rank:	PSO	GA	SGA	PGPHEA	HPSOM
Average	2.93	4.14	1.57	2.07	3.79
Overall	3	5	1	2	4

**Fig 9:**
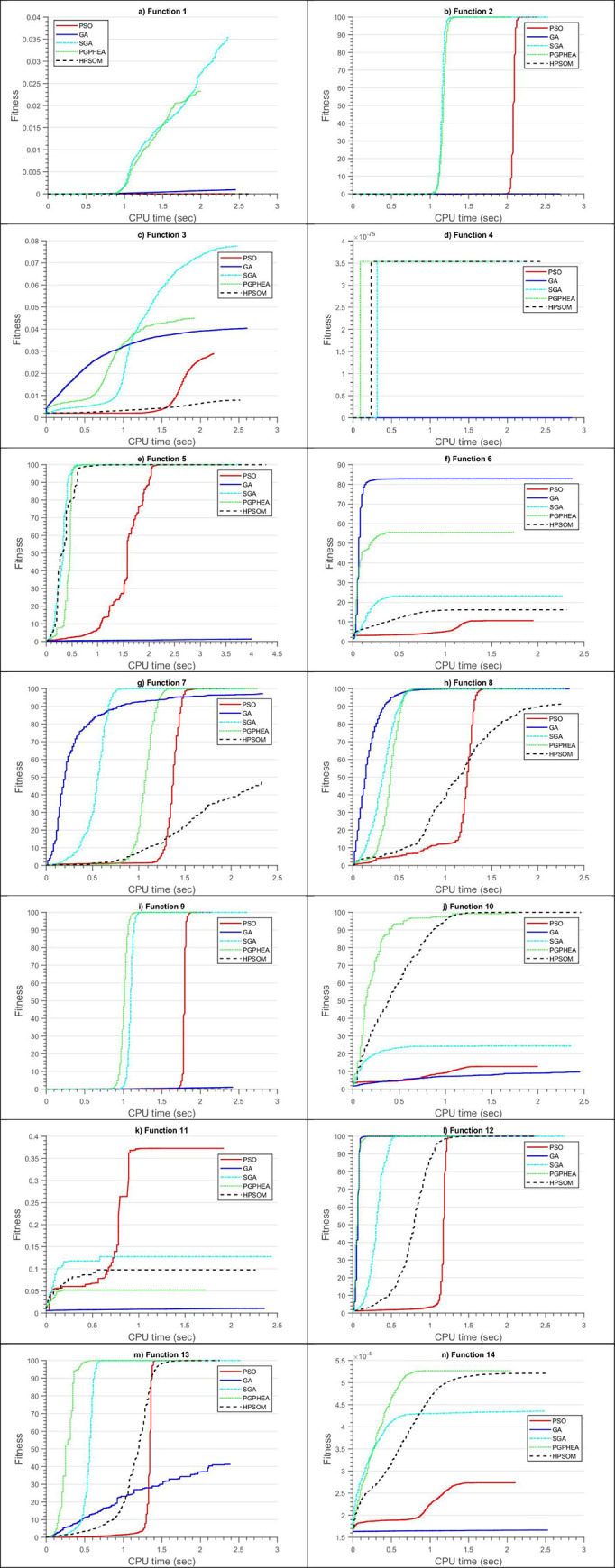
Graphical representation of the evolution of fitness with CPU time spent for Set A continuous problems (*f*_1_-*f*_14_).

The observation that SGA provided better results for a given number of objective function evaluations does not necessarily mean that it is the most computationally efficient. This is because the other calculations in each algorithm (e.g. generation of random numbers, algebraic operations of GA crossovers and PSO velocities) and their relative proportions result in differences in computational cost among the algorithms. Moreover, an algorithm, although not achieving the minimum error, may approach the global optimum to a practically adequate degree faster than the algorithm that yields the least error. To investigate this aspect, the convergence speed, i.e., the evolution of the average fitness (of the 100 runs) with CPU time (as reported by Matlab), is plotted in Fig 9. The fitness is defined herein as

$$fitness=\frac{1}{error_{iter}+0.01}$$ (9)

The perfect fitness is equal to 100, meaning that the error is equal to zero.

In unconstrained optimization problems *f*_2_ & *f*_5_ and the constrained problems *f*_7_ & *f*_8_, SGA reaches fitness larger than 99 faster than the rest of the algorithms. GA in general is much slower, but its convergence rate is steady (e.g. *f*_3_, *f*_7_, *f*_10_ and *f*_13_). Oppositely, PSO generally attains high fitness values much faster than GA once a breakthrough from local minima is made (e.g. *f*_2_, *f*_5_, *f*_9_, *f*_11_, *f*_13_)_._ It is interesting to note that PGPHEA and SGA have fitness evolution patterns (curve shape) that are similar to those of PSO, but in most cases overcome local minima and reach high fitness levels at a shorter CPU time.

#### 4.1.2 Set B

The second set comprises the 29 single-objective continuous problems (F1,F3-F30) of the CEC 2017 benchmark suite [33, 34]. This suite contains unimodal, multimodal, hybrid, and composition functions. The optimization algorithms are tested with the dimension *D* for all benchmarks set equal to 50. The population size and algorithmic parameters are the same as in the case of the Set A problems presented in the previous section, with the difference that the termination criterion is set to 500100 function solution (5000 steps for a population of 100).

**Fig 10:**
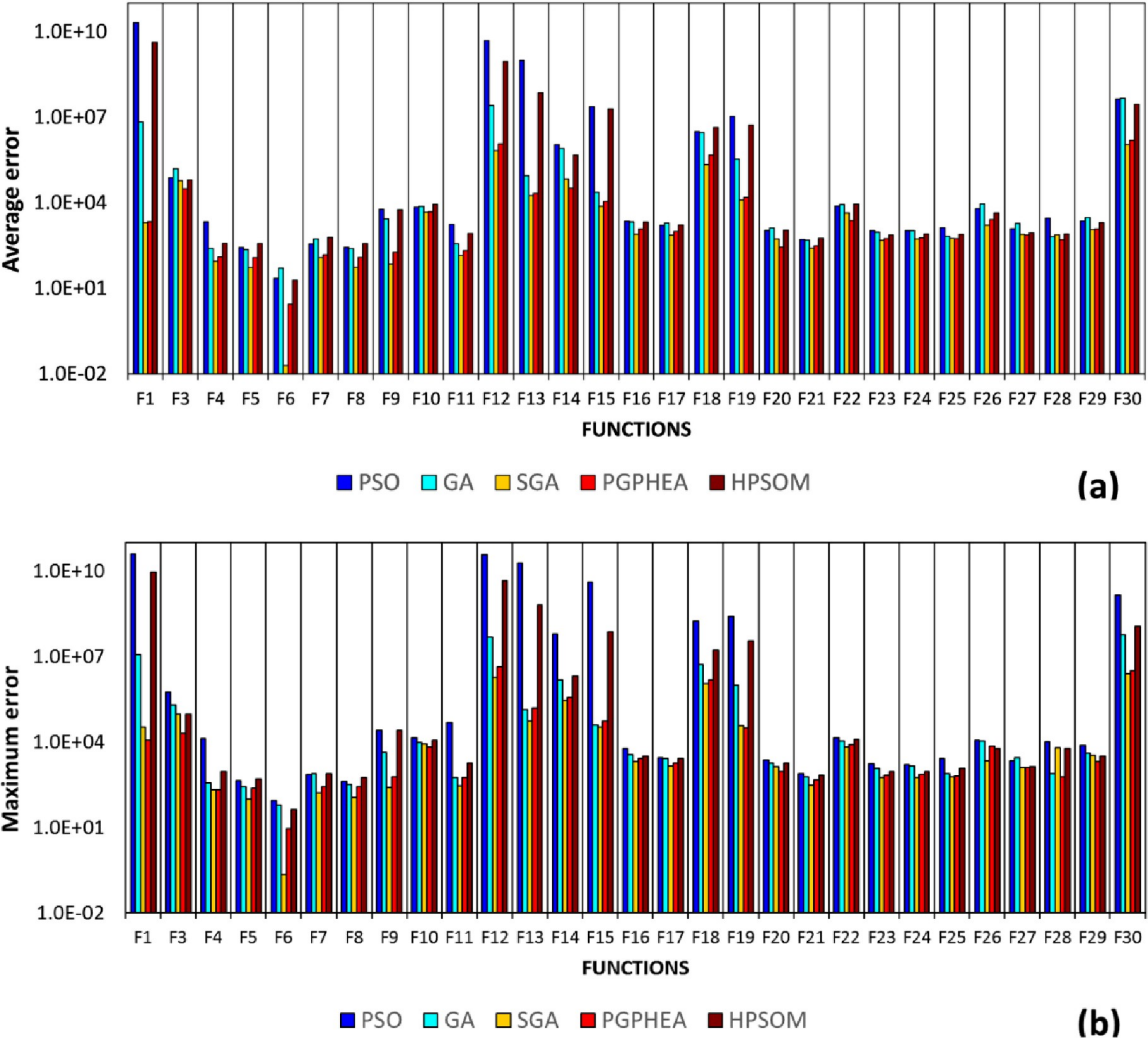
Comparison of algorithmic performance in solving continuous problems of Set B (CEC 2017): a) average error, b) maximum error in predicting the global optimum.

The average error and maximum error from the 100 runs are shown in Tables 7 and 8, respectively, where grey shading marks the best performing algorithms in each case. The results are also compared graphically in Fig 10.

**Table 7:** Comparison of average error in finding the global optimum for CEC 2017 problems.

Function	PSO	GA	SGA	PGPHEA	HPSOM
F1	2.03E+10	6.80E+06	2.01E+03	2.16E+03	4.18E+09
F3	7.54E+04	1.57E+05	5.92E+04	3.09E+04	6.37E+04
F4	2.13E+03	2.49E+02	8.93E+01	1.25E+02	3.81E+02
F5	2.68E+02	2.27E+02	5.32E+01	1.19E+02	3.73E+02
F6	2.23E+01	5.10E+01	1.97E-02	2.75E+00	1.93E+01
F7	3.65E+02	5.36E+02	1.20E+02	1.49E+02	6.07E+02
F8	2.74E+02	2.47E+02	5.39E+01	1.21E+02	3.74E+02
F9	5.95E+03	2.73E+03	7.09E+01	1.86E+02	5.61E+03
F10	7.03E+03	7.44E+03	4.58E+03	4.87E+03	8.92E+03
F11	1.74E+03	3.68E+02	1.41E+02	2.09E+02	8.46E+02
F12	4.86E+09	2.56E+07	6.68E+05	1.16E+06	9.03E+08
F13	9.79E+08	8.89E+04	1.79E+04	2.12E+04	7.15E+07
F14	1.08E+06	8.02E+05	6.74E+04	3.25E+04	4.64E+05
F15	2.34E+07	2.34E+04	7.60E+03	1.10E+04	1.94E+07
F16	2.24E+03	2.11E+03	7.86E+02	1.19E+03	2.08E+03
F17	1.62E+03	1.96E+03	7.34E+02	9.79E+02	1.67E+03
F18	3.15E+06	2.91E+06	2.16E+05	4.64E+05	4.35E+06
F19	1.05E+07	3.42E+05	1.26E+04	1.56E+04	5.27E+06
F20	1.06E+03	1.31E+03	5.38E+02	2.77E+02	1.08E+03
F21	5.10E+02	4.87E+02	2.53E+02	3.10E+02	5.67E+02
F22	7.73E+03	8.72E+03	4.37E+03	2.36E+03	9.00E+03
F23	1.04E+03	9.36E+02	4.82E+02	5.54E+02	7.42E+02
F24	1.05E+03	1.05E+03	5.33E+02	5.86E+02	8.06E+02
F25	1.32E+03	6.69E+02	5.63E+02	5.56E+02	7.76E+02
F26	6.22E+03	9.15E+03	1.65E+03	2.59E+03	4.39E+03
F27	1.21E+03	1.89E+03	7.84E+02	7.49E+02	8.77E+02
F28	2.88E+03	6.44E+02	7.61E+02	5.05E+02	8.05E+02
F29	2.27E+03	3.05E+03	1.13E+03	1.19E+03	2.03E+03
F30	4.31E+07	4.55E+07	1.08E+06	1.56E+06	2.84E+07
**W/T/L**	**0/0/29**	**0/0/29**	**22/0/7**	**7/0/22**	**0/0/29**
**OE**	**0.00%**	**0.00%**	**75.86%**	**24.14%**	**0.00%**

**Table 8:** Comparison of maximum error in finding global optimum for CEC 2017 problems.

Function	PSO	GA	SGA	PGPHEA	HPSOM
F1	3.91E+10	1.16E+07	3.18E+04	1.11E+04	9.00E+09
F3	5.57E+05	1.99E+05	9.36E+04	2.00E+04	9.26E+04
F4	1.27E+04	3.55E+02	2.04E+02	2.13E+02	9.20E+02
F5	4.28E+02	2.74E+02	1.02E+02	2.34E+02	4.96E+02
F6	8.59E+01	6.06E+01	2.16E-01	8.76E+00	4.11E+01
F7	6.95E+02	7.42E+02	1.64E+02	2.61E+02	7.56E+02
F8	4.10E+02	3.19E+02	1.09E+02	2.71E+02	5.50E+02
F9	2.48E+04	4.36E+03	2.45E+02	5.85E+02	2.57E+04
F10	1.34E+04	9.59E+03	8.53E+03	6.54E+03	1.18E+04
F11	4.82E+04	5.58E+02	2.90E+02	5.69E+02	1.76E+03
F12	3.67E+10	4.76E+07	1.78E+06	4.22E+06	4.57E+09
F13	1.92E+10	1.36E+05	5.45E+04	1.53E+05	6.36E+08
F14	6.20E+07	1.53E+06	2.75E+05	3.54E+05	1.98E+06
F15	4.05E+09	3.94E+04	3.25E+04	5.24E+04	7.17E+07
F16	5.78E+03	3.52E+03	2.00E+03	2.54E+03	3.07E+03
F17	2.70E+03	2.60E+03	1.39E+03	1.76E+03	2.58E+03
F18	1.77E+08	5.11E+06	1.10E+06	1.52E+06	1.63E+07
F19	2.49E+08	9.75E+05	3.68E+04	3.11E+04	3.40E+07
F20	2.26E+03	1.85E+03	1.36E+03	9.34E+02	1.79E+03
F21	7.41E+02	5.93E+02	2.94E+02	4.73E+02	6.51E+02
F22	1.39E+04	1.08E+04	6.65E+03	7.84E+03	1.25E+04
F23	1.69E+03	1.19E+03	5.53E+02	6.67E+02	9.11E+02
F24	1.55E+03	1.42E+03	5.61E+02	6.95E+02	8.98E+02
F25	2.61E+03	7.43E+02	6.11E+02	6.26E+02	1.14E+03
F26	1.16E+04	1.09E+04	2.10E+03	7.16E+03	5.80E+03
F27	2.19E+03	2.75E+03	1.23E+03	1.23E+03	1.34E+03
F28	9.99E+03	7.42E+02	6.26E+03	5.92E+02	5.71E+03
F29	7.51E+03	4.12E+03	3.28E+03	2.00E+03	3.21E+03
F30	1.38E+09	5.64E+07	2.44E+06	3.06E+06	1.13E+08
**W/T/L**	**0/0/29**	**0/0/29**	**21/0/8**	**8/0/21**	**0/0/29**
**OE**	**0.00%**	**0.00%**	**72.41%**	**27.59%**	**0.00%**

It can be seen that SGA exhibits the best performance, followed by PGPHEA (Table 9). SGA achieves the smallest average and maximum errors for 22 and 21 out of the 29 functions, respectively (Table 7). Moreover, the Wilcoxon test (Table 10) as well as the Friedman test (with p-value = 6.8896e-20) indicate that there are significant statistical differences in the performance of the examined algorithms. In the case of CEC 2017 suite, the parent algorithms GA and PSO cannot provide better results than the hybrid algorithms SGA and PGPHEA in any of the benchmark functions. The evolution of the global best value (average of 100 runs) with CPU time for the 29 benchmark problems can be found in S1 Fig in S1 File. It can be seen that in a number of problems (e.g. F14,F18,F26,F27) SGA is ahead from the other algorithms from the beginning of the solution process. However, in most of the cases, SGA gains an edge in later stages, often with a sharp improvement of the global best, indicating an escape from a sub-optimal solution (e.g. F5,F7,F8,F10,F16,F17,F20,F21,F23,F24).

**Table 9:** Algorithm Ranking for CEC 2017 problems.

Rank:	PSO	GA	SGA	PGPHEA	HPSOM
Average	4.72	3.52	1.28	1.76	3.72
Overall	5	3	1	2	4

**Table 10:** Wilcoxon signed-rank-test (*p* ≥ 0.05) for average error/ in CEC 2017 problems.

SGA vs.	PSO	GA	PGPHEA	HPSOM
p-value	1.86E-09	9.31E-09	1.01E-02	1.86E-09
Significant	Yes	Yes	Yes	Yes

### 4.2 Discrete problems

The algorithms were tested for 9 cases of the traveling salesman problem (TSP) of the library TSPLIB [38]. In order to render the PSO algorithm able to solve discrete problems, a PSO adaptation is necessary, e.g. [39–45]. In this paper, we adopt the mapping proposed in [41] according to which each feasible solution (permutation) is linked to a vector (particle position) **x** = {*x*_1_,*x*_2_,…,*x*_*D*_} the elements of which are numbers signifying the priorities of the 1,…, *D* cities. For example, if we have a six-variable permutation problem [A B C D E F], each letter will match the corresponding position, i.e., *x*_1_ is city’s “A” priority. A particle vector {0.91, 0.72, 0.87, 0.12, 0.61, 0.89} would result in the following permutation [A F C B E D].

Unlike in the continuous problems (in which case arithmetic crossover was employed), for the crossover operations of the GA (either as standalone algorithm or as a component in the hybrid algorithms), the order crossover approach of [46] was used. Each new individual follows the ‘travel’ order of one parent until a random point from which it starts following the second parent’s ‘travel’ order by skipping the visited cities. For example, if the travel order of the first parent is [A C B F D E] and that of the 2^nd^ parent is [E C B A D F] with the crossover point being the 4^th^ city, the offspring’s ‘travel’ order is [A C B F] + [E D] = [A C B F E D]. On the other hand, the mutation is a simple swap between two cities priority order. For an individual’s order [A F C B E D], a mutation (1,5) would result in the new order [E F C B A D].

The parameters for each algorithm were optimized through trial runs of the nine TSP benchmarks. The parameters *c*_1_ and *c*_2_ (Eq 4) are equal to 2 in all algorithms involving PSO operations. The inertial factor *w* is set equal to a fixed value (no “damping”) of 0.01 for SGA, PGPHEA and HPSOM, while for PSO it is set to decrease linearly (Eq 6) with *w*_max_ = 1 and *w*_min_ = 0.4. The overall population size was *M* = 20 for all algorithms. For GA (either as standalone algorithm or as a component in the hybrid algorithms), an elite group of 20% of the population is isolated in order to undergo elite crossover, 30% undergoes mutation and 50% crossover. For PGPHEA, it was found that its best performance is achieved with *n*_e_ = 1, i.e. the exchange between PSO and GA sub-populations happens after every iteration. For HPSOM, 40% of the population is set to undergo mutation. In SGA, every *n*_g_ = 2 iterations of GA (external loop), 25% of the population is randomly selected to undergo *n*_p_ = 5 PSO iterations (internal loop). As a termination criterion, a maximum number of the route distance calculations equal to 40020 is considered.

**Fig 11:**
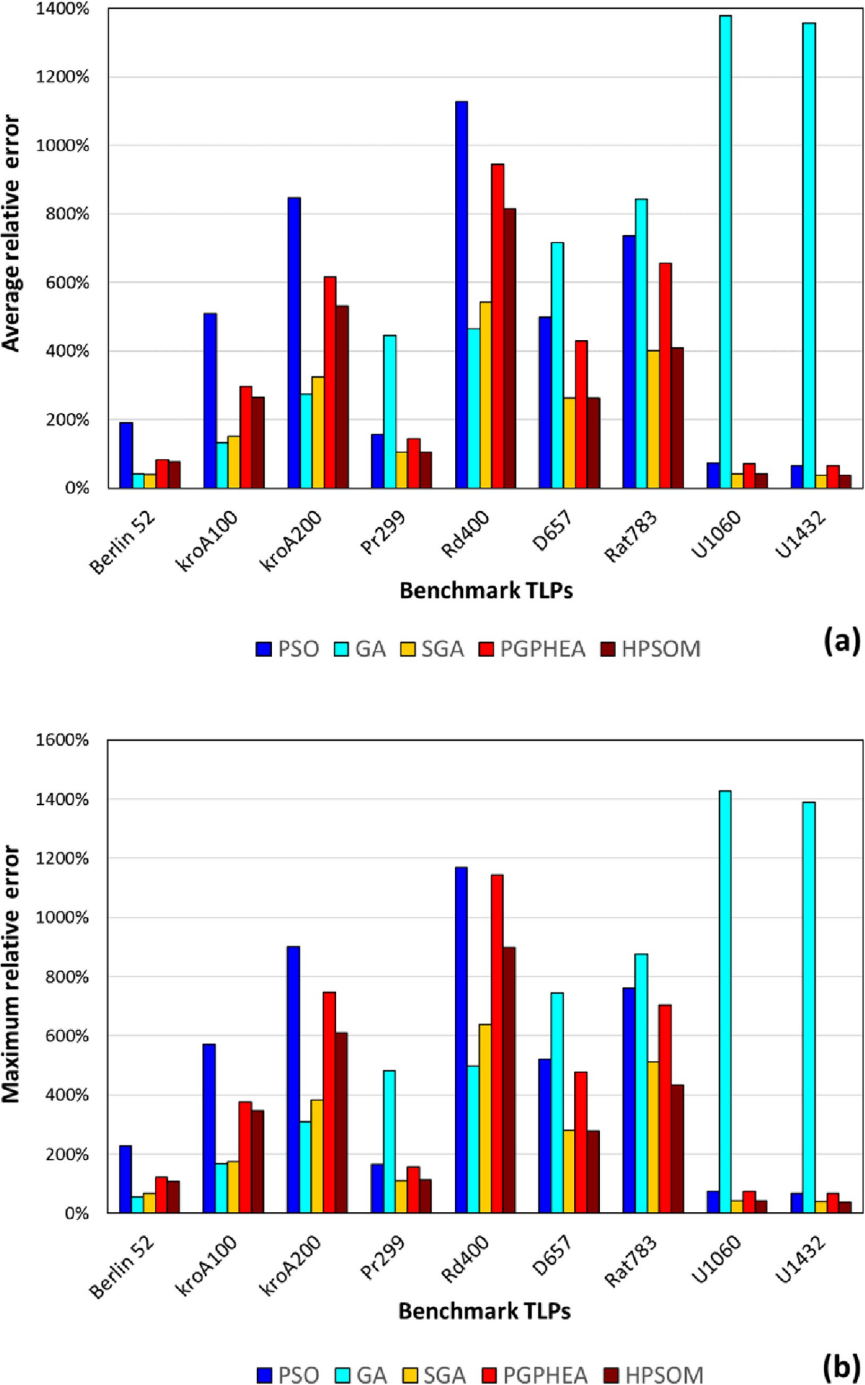
Comparison of algorithmic performance in solving TLP: a) average relative error, b) maximum relative error in predicting the optimal distance.

Each problem was solved 100 times with each algorithm, and the average and maximum relative errors in the calculated optimal distance are shown in Tables 11 and 12, respectively, and in Fig 11.

**Table 11:** Comparison of average relative distance error for traveling salesman problems.

TSP problem	Global optimum	PSO	GA	SGA	PGPHEA	HPSOM
Berlin 52	7542	190.48%	41.50%	39.13%	82.51%	76.58%
kroA100	21282	508.99%	132.81%	150.45%	296.75%	264.48%
kroA200	29368	847.92%	275.24%	323.45%	616.20%	531.85%
Pr299	48191	157.80%	445.82%	104.81%	144.69%	104.75%
Rd400	15281	1127.11%	465.63%	543.77%	945.53%	815.89%
D657	48912	498.92%	716.75%	261.65%	429.28%	262.68%
Rat783	8806	736.01%	843.86%	400.84%	657.38%	409.59%
U1060	224094	72.52%	1378.71%	40.64%	70.82%	40.76%
U1432	152870	65.88%	1357.67%	36.97%	65.70%	37.01%
**Average**		**467.29%**	**628.67%**	**211.30%**	**367.65%**	**282.62%**
**W/T/L**		**0/0/9**	**3/0/6**	**5/0/4**	**0/0/9**	**1/0/8**
**OE**		**0%**	**33.3%**	**55.5%**	**0%**	**11.1%**

**Table 12:** Comparison of maximum distance error for traveling salesman problems.

TSP problem	Global optimum	PSO	GA	SGA	PGPHEA	HPSOM
Berlin 52	7542	227.82%	57.03%	68.32%	122.24%	108.55%
kroA100	21282	572.03%	167.30%	175.06%	375.62%	346.53%
kroA200	29368	900.74%	311.09%	382.64%	747.02%	611.32%
Pr299	48191	165.93%	481.81%	111.34%	156.51%	113.36%
Rd400	15281	1169.48%	497.35%	637.73%	1144.23%	898.46%
D657	48912	521.44%	744.82%	281.03%	477.64%	277.83%
Rat783	8806	762.23%	876.45%	511.67%	704.25%	433.64%
U1060	224094	75.30%	1426.88%	42.69%	74.44%	43.08%
U1432	152870	68.16%	1388.15%	38.92%	67.39%	38.74%
**Average**		**495.90%**	**661.21%**	**249.93%**	**429.93%**	**319.06%**
**W/T/L**		**0/0/9**	**4/0/5**	**2/0/7**	**0/0/9**	**3/0/6**
**OE**		**0%**	**44.4%**	**22.2%**	**0%**	**33.3%**

It can be seen that, in the case of TSPs, the GA and PSO algorithms along with the three hybrids have difficulty in finding the global optimum at the prescribed maximum number of objective function evaluations (termination criterion). Nonetheless, a distinct pattern emerges, different than that in the continuous benchmark problems. In overall, PSO appears to be the worst performing algorithm by a significant margin in many cases, and its hybridization results in an improved performance to various degrees (Fig 11). GA is underperforming particularly for the problems containing large number of cities. Nonetheless, it is worth to note that GA is the best performing algorithm for 3 out of the 9 TSP benchmarks. SGA outperforms the other algorithms in 5 out of 9, while HPSOM in 1 out of 9 (Table 11, Fig 11A), with a Friedman test *p*-value equal to 9.86E-05, indicating significant differences across all algorithms. Although SGA’s ranks first on average (Table 14), according to the Wilcoxon test (Table 13) it can’t be claimed SGA can provide better results than GA with 0.05 significance. Nonetheless, it should be noted that, in terms of maximum relative error, HPSOM seems to have an edge over SGA (Table 12, Fig 11B). Finally, it is worth mentioning that HPSOM provides better results compared to PGPHEA, in contrast to what was observed in the case of continuous problems (Table 14).

**Table 13:** Wilcoxon signed-rank-test (*p* ≥ 0.05) for average error in continuous problems.

SGA vs.	PSO	GA	PGPHEA	HPSOM
p-value	1.95E-03	6.45E-02	1.95E-03	1.56E-02
Significant	Yes	No	Yes	Yes

**Table 14:** Discrete problem algorithm ranking.

Rank:	PSO	GA	SGA	PGPHEA	HPSOM
Average	4.44	3.33	1.44	3.44	2.33
Overall	5	3	1	4	2

## 5. Conclusions

A new hybrid heuristic algorithm, named Swarming Genetic Algorithm (SGA) was proposed, nesting Particle Swarm Optimization (PSO) loops inside the Genetic Algorithm (GA), in order to enhance the efficiency of the latter in attaining fast the global optimum. The aim was to counter each algorithm’s shortcomings by taking advantage of the exploitation capabilities of PSO and combine them with the well-known exploration prowess of GA. In order to assess the performance of the new hybrid algorithm, two sets of continuous and one set of discrete (traveling salesman) benchmark problems were examined and comparisons were made against GA, PSO and two existing hybrid algorithms. The results showed that SGA has in overall significantly better performance than PSO and GA in terms of accuracy, in both continuous and discrete problems.

## Supporting information

S1 File (PDF)

S2 File Data of plots of Fig 9: Fitness vs CPU time. (XLSX)

S3 File Data of plots of S1 Fig: Global best value vs CPU time. (XLSX)
