# Supplementary material for: Swarming genetic algorithm: A nested fully coupled hybrid of genetic algorithm and particle swarm optimization
Source: PLoS One. 2022 Sep 23;17(9):e0275094. doi: 10.1371/journal.pone.0275094 (PMC9506650; doi:10.1371/journal.pone.0275094)
Supplement: S1 File — (PDF) [file pone.0275094.s001.pdf]

S1. APPENDIX

Table S1. Effect of inertial parameters in the performance of PSO, PGPHEA and HPSOM.

CEC2017 Function	PSO		PGPHEA		HPSOM	
	w=2, w_{min}=0.01	w=1, w_{min}=0.001	w=2, w_{min}=0.01	w=1, w_{min}=0.001	w=2, w_{min}=0.01	w=1, w_{min}=0.001
F1	1.98E+10	2.03E+10	2.16E+03	1778.134	4.51E+09	4.18E+09
F3	2.18E+05	75395.57	3.09E+04	9401.333	1.07E+05	6.37E+04
F4	4.08E+03	2130.081	1.25E+02	100.9305	4.59E+02	3.81E+02
F5	2.66E+02	267.9317	1.19E+02	141.7486	4.13E+02	3.73E+02
F6	3.33E+01	22.27109	2.75E+00	1.627532	2.61E+01	1.93E+01
F7	3.78E+02	364.8595	1.49E+02	171.572	6.30E+02	6.07E+02
F8	2.76E+02	273.8523	1.21E+02	138.3212	4.11E+02	3.74E+02
F9	7.97E+03	5946.714	1.86E+02	297.245	7.08E+03	5.61E+03
F10	1.11E+04	7032.559	4.87E+03	4591.19	1.01E+04	8.92E+03
F11	7.79E+03	1743.236	2.09E+02	231.9872	1.01E+03	8.46E+02
F12	1.16E+10	4.86E+09	1.16E+06	679951.9	1.18E+09	9.03E+08
F13	4.32E+09	9.79E+08	2.12E+04	23840.56	1.05E+08	7.15E+07
F14	1.07E+07	1080493	3.25E+04	44159.49	6.95E+05	4.64E+05
F15	5.18E+08	23434315	1.10E+04	11236.55	3.59E+07	1.94E+07
F16	3.36E+03	2237.17	1.19E+03	1441.365	2.55E+03	2.08E+03
F17	1.75E+03	1616.465	9.79E+02	1156.943	1.87E+03	1.67E+03
F18	3.02E+07	3151556	4.64E+05	237370.3	4.97E+06	4.35E+06
F19	2.79E+07	10484467	1.56E+04	14102.1	8.64E+06	5.27E+06
F20	1.63E+03	1058.03	2.77E+02	399.4825	1.43E+03	1.08E+03
F21	5.47E+02	509.8796	3.10E+02	319.6457	6.12E+02	5.67E+02
F22	1.14E+04	7730.576	2.36E+03	3175.629	1.03E+04	9.00E+03
F23	1.38E+03	1041.914	5.54E+02	569.6088	8.06E+02	7.42E+02
F24	1.24E+03	1054.632	5.86E+02	608.5232	8.67E+02	8.06E+02
F25	1.28E+03	1324.766	5.56E+02	556.7337	8.10E+02	7.76E+02
F26	9.71E+03	6223.2	2.59E+03	2727.329	4.97E+03	4.39E+03
F27	1.48E+03	1214.766	7.49E+02	800.3936	9.15E+02	8.77E+02
F28	4.61E+03	2878.702	5.05E+02	498.2927	2.90E+03	8.05E+02
F29	4.22E+03	2270.921	1.19E+03	1217.243	3.00E+03	2.03E+03
F30	1.82E+08	43090432	1.56E+06	1664385	4.09E+07	2.84E+07
**W/T/L**	**3/0/26**	**26/0/3**	**20/0/9**	**9/0/20**	**0/0/29**	**29/0/0**

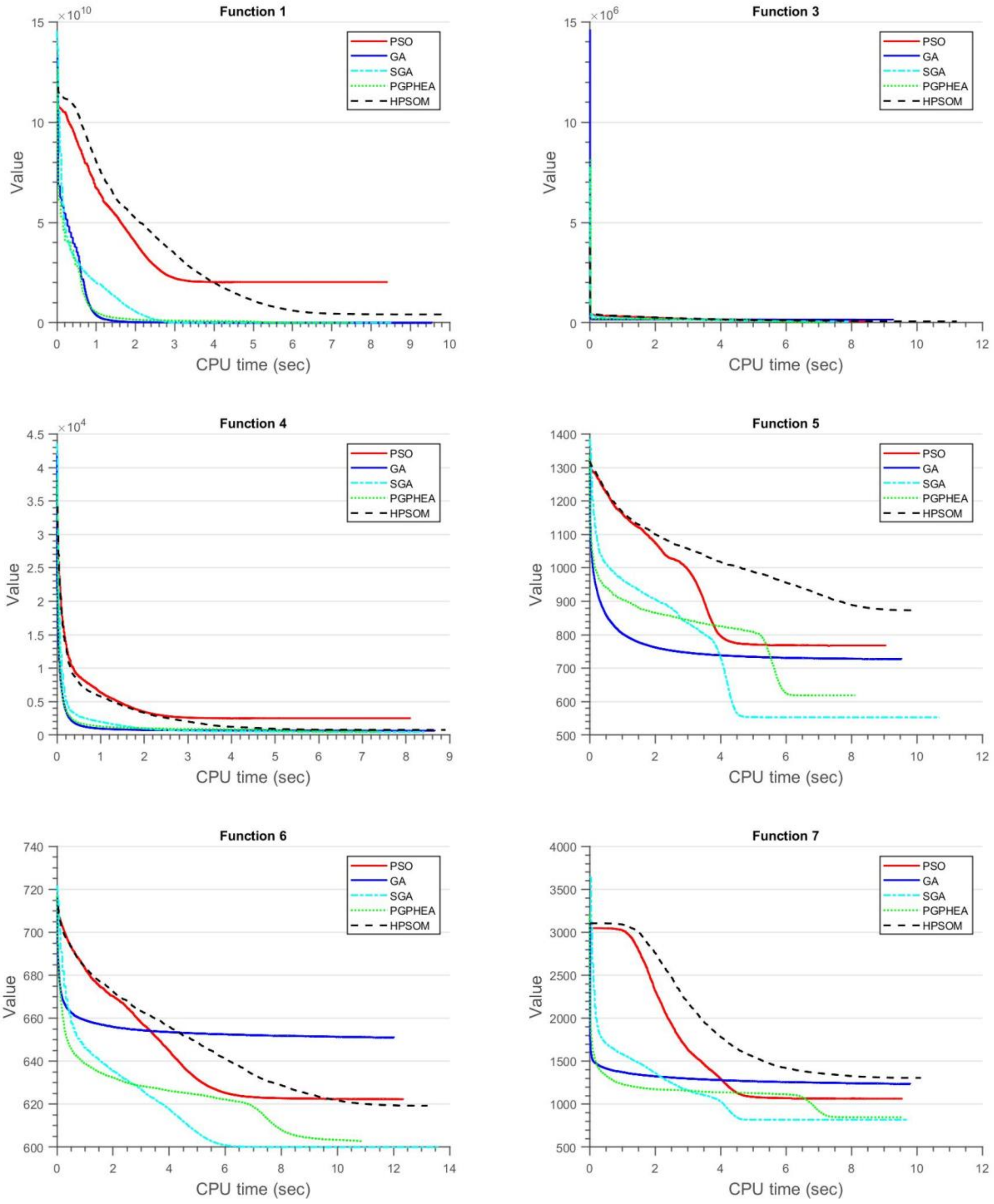

Figure S1. Evolution of best value with CPU time for the CEC 2017 benchmark problems.

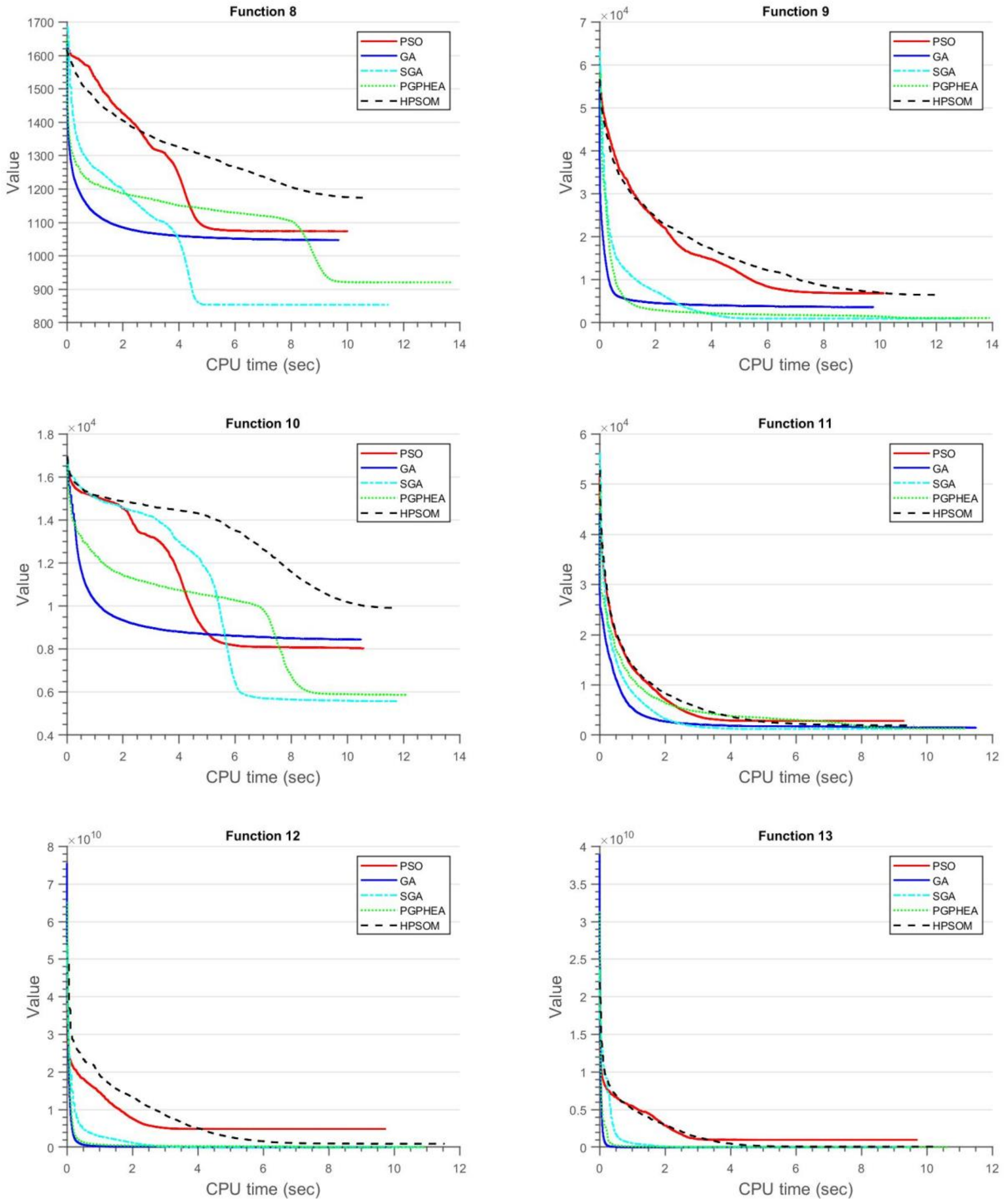

Figure S1. (continued)

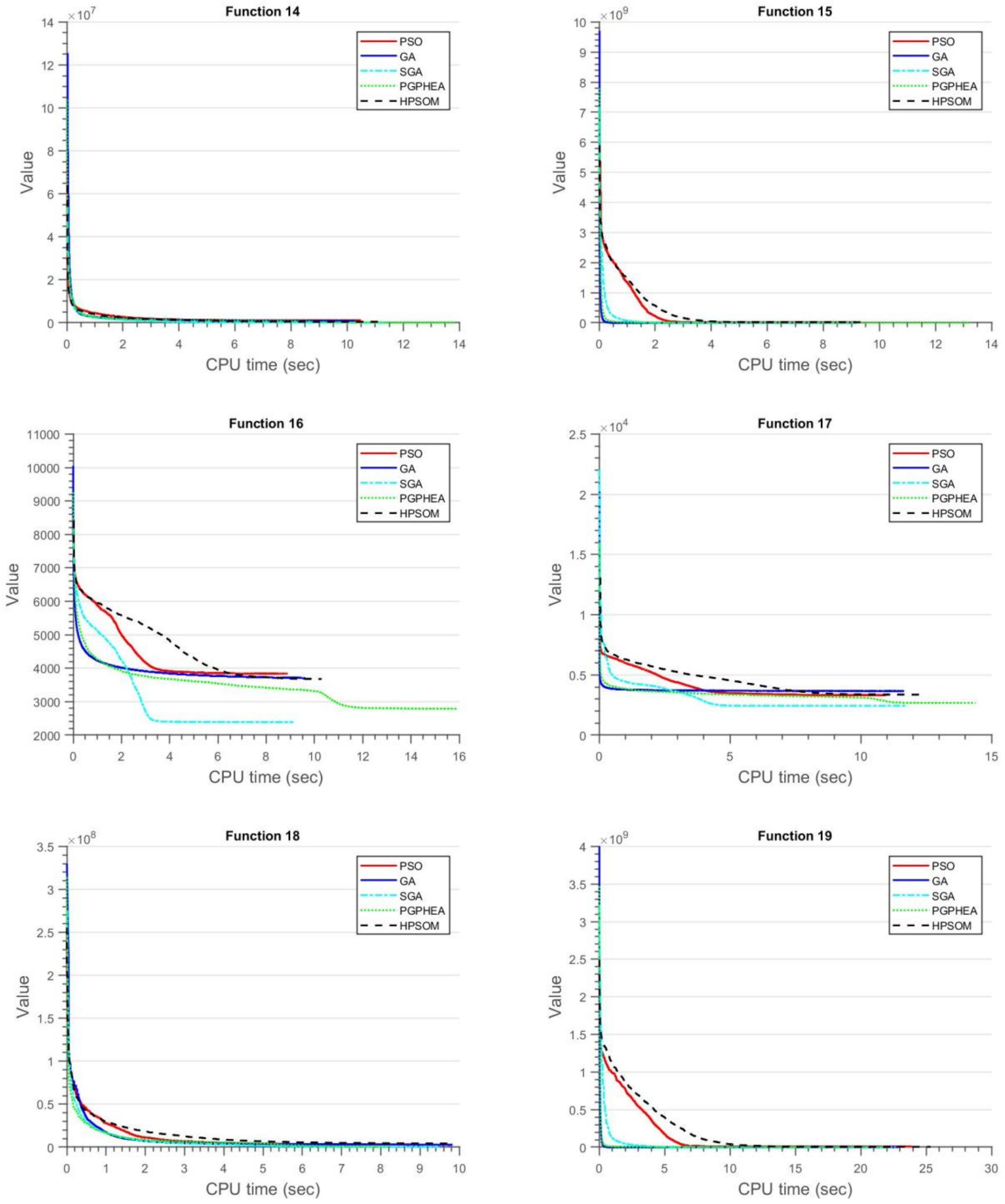

Figure S1. (continued)

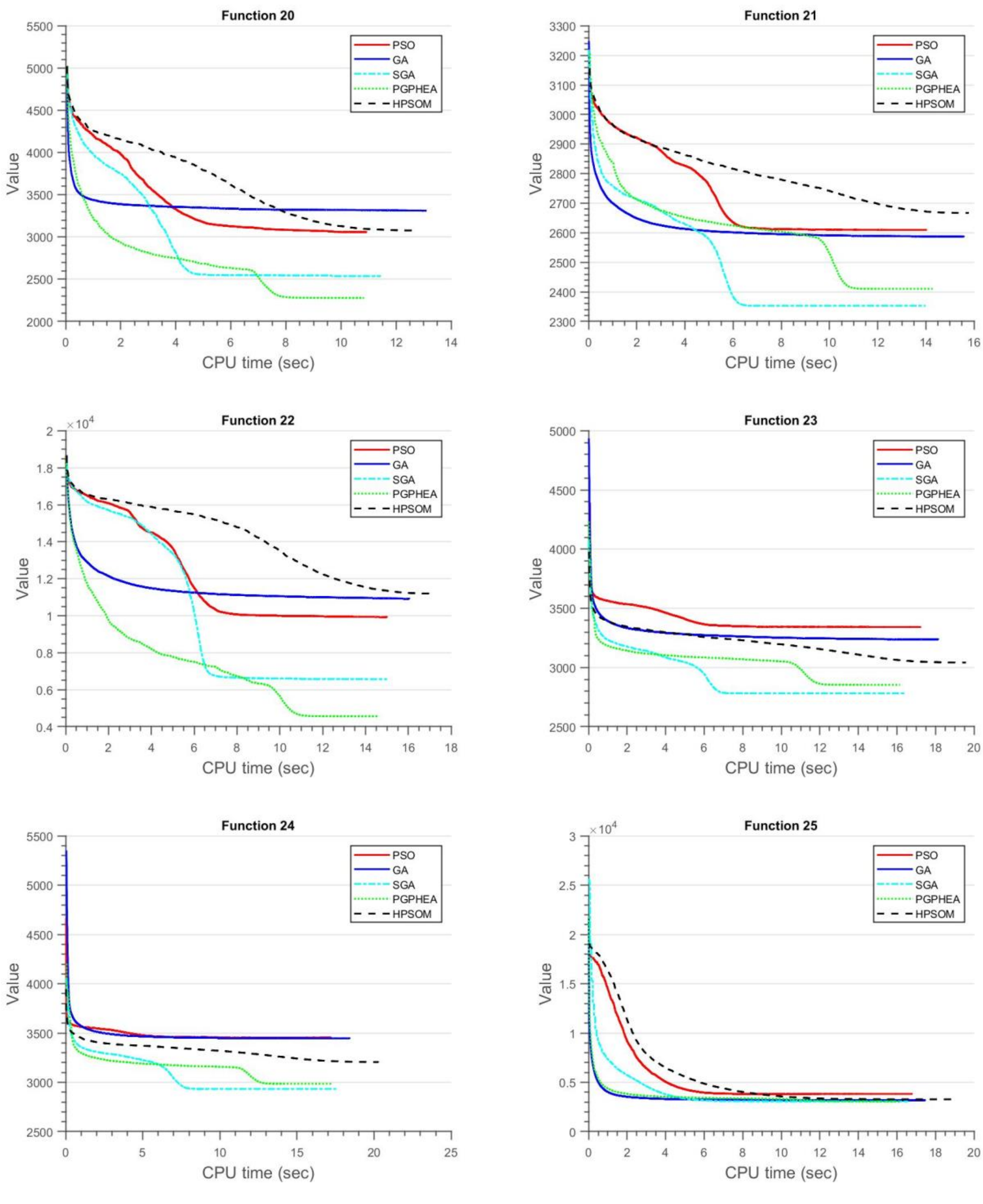

Figure S1. (continued)

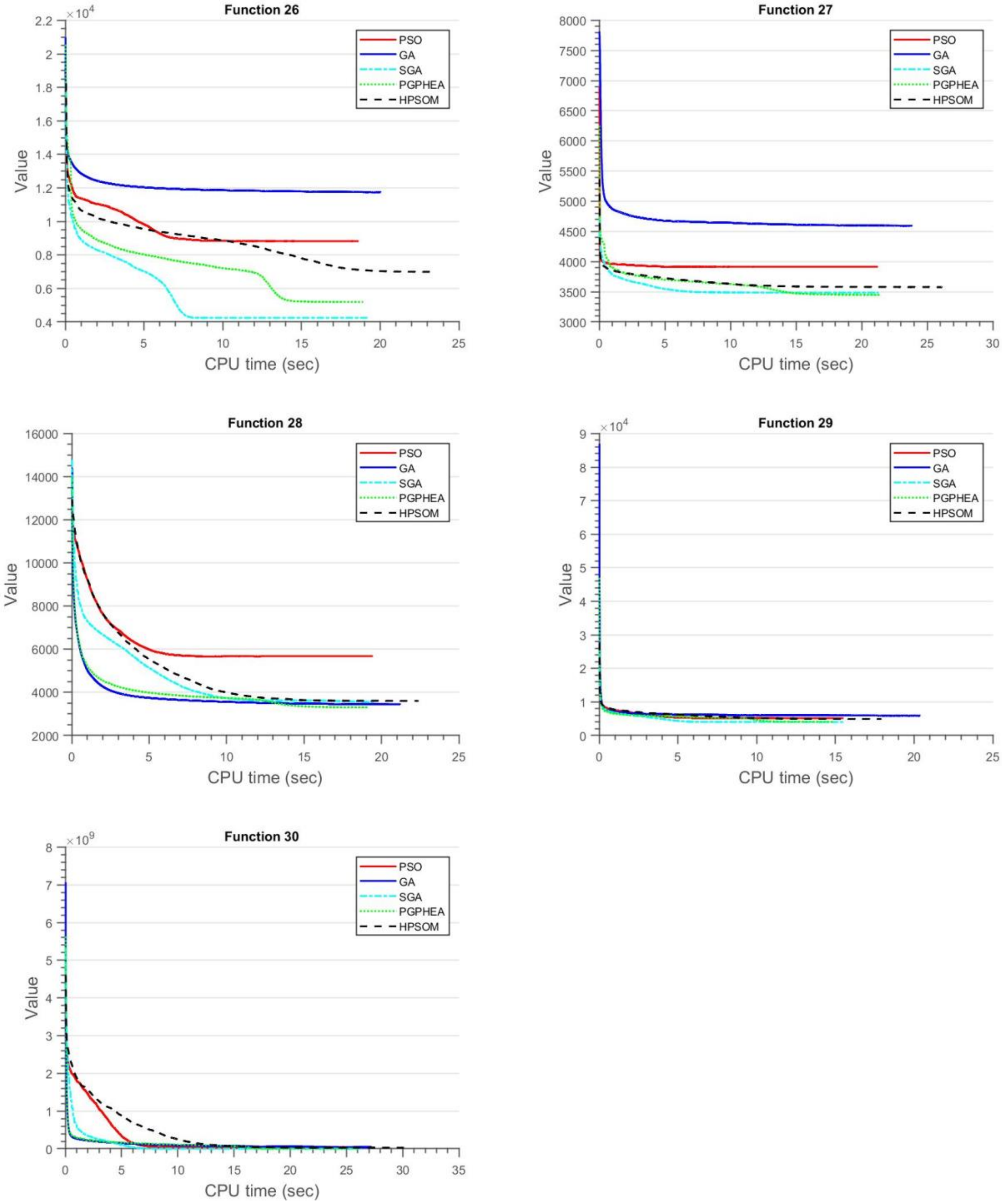

Figure S1. (continued)
